# Supplementary material for: Cultural adaptation of a UK evidence-based problem-solving intervention to support Polish prisoners at risk of suicidal behaviour: a cross-sectional survey using an Ecological Validity Model
Source: BMJ Open. 2023 Jul 9;13(7):e069252. doi: 10.1136/bmjopen-2022-069252 (PMC10335472; doi:10.1136/bmjopen-2022-069252)
Supplement: Supplementary data [file bmjopen-2022-069252supp001.pdf]

Supplementary File

Session one: Role play example – demonstrating how a negative attitude can affect the outcome of a problem

- Facilitator introduce the first role-play example to show about people behave and react to a situation can vary depending upon how they feel:

'That you are on your way to an interview in your car and you suddenly realised that you have t wo but two flat tyres. You suspect that a neighbour has let your tyres down. All dressed up in a suit and tie. You have to stop I the middle of the road blocking the traffic which grinds to a halt behind you which starts causing chaos.' A bus stop is near by. Someone in another car stops and laughs at you – pulls over the stops you go over to them…

- Facilitators to demonstrate the scenario

- Facilitator to question the group:
    - How did the driver feel about the situation?
    - What was there attitude like?
    - Did the incident end in a positive way?
    - What could they have done differently/said?

Facilitator to encourage the group to mimic the role-play exercise so…

- Ok so I want you to get into pairs. One person be the driver who approaches the person who has laughed at them. I want you to start your conversation telling the person how you feel what your problem is and see how they respond.
- When we have done this we are going to go around the room and talk about what happened in each person's situation – was the problem resolved- did the person help – did they get to the interview or not?
- Then repeat the exercise and role play it with a positive attitude. The exercise shows that if you do things in the right way you can often get a more satisfactory outcome.

On a flip chart and with the whole group the facilitator uses the example to demonstrate the problem-solving steps.

Steps in the model:

Step1: Getting the right attitude

Step 2:Reflect and recognise triggers – facilitator notes that if people reflect on previous incidents that didn't turn out as anticipated this can help to identify how they felt at the time, what they thought and how they behaved. Recognising these triggers can play an important role in behaviour change.

Step 3: Define the problem clearly – often we don't have all the information we need to resolve a situation and sometimes you might use these skills to help support how someone feels to enable them to cope better with a situation. Facilitator to prompt – what information did the person does not have in the car scenario. Using what/where/when/why type questions can help to elicit this information in more detail. This specific information helps someone to explore the crux of the problem. To use the problem-solving skills effectively a problem needs to be clearly defined. For example, 'I have a problem with my wife' isn't a problem that is sufficiently defined to be able to use the skills.

Step 4 Brainstorming solutions – Once we have as much information as possible and a clear problem definition, we encourage people to think of as many different solutions (good or bad) to help promote ideas that the person might want to try out. In this model of problem solving, it is important that the person comes up with their own ideas (see the list of suggestions in Appendix 1) if someone requires some support to do this.

Step 5 Decision making – For each solution we then make a list with the possible pros and cons of each idea. It is good to go through the 'not so positive ideas' as it allows someone to explore what might go wrong is they choose an option that is less than favourable. This process helps someone to shortlist the ideas to two or three that they wish to pursue and take forwards into an action plan.

Step 6 Make a plan -Then we think about making a plan – this can be a different plan for everybody [the right plan for one person might not be the case for someone else]. Write down your plan – what would you do? What is your plan? The more specific the plan is the more likely it will work. In what order are you going to do the tasks? When are you going to do each task? Do you need some help or support to complete the task?

Step 7 Review – Problem-solving is an iterative process and not always does it work for many logistical reasons. It is therefore sensible to support an individual to review what happened. Did your plan work? What went wrong? Using the skills again to produce another alternative and try again using the skills. The more someone practicing the skills the better they get at using them.

Male Case Study Example:

James is 22 years old. He is one of six siblings and lives with his father and stepmother. James's relationship with his parents was troubled from a young age and his father would come home drunk and beat James. His stepmother found it difficult to deal with James's aggressive emotional outbursts and he was excluded from school at age 11 for poor behaviour and angry outbursts.

James started to mix with a gang of older boys who were known in the area for committing petty crimes. He became involved in drugs at age 13 years and was caught by the police for burglary when he was 16 years old. He also had a series of relationships with older women which led to a number of pregnancies resulting in two sons and a new baby. James's stepmother was unable to control his behaviour and did not want him in the house anymore so James was asked to leave.

James went to stay with a friend but soon ended up living in a hostel. He found it difficult to get a job and ended up stealing to support his drug habit. His physical and mental health deteriorated and he no longer took care of himself. One day he took drugs and alcohol and ended up in the Accident and Emergency department at the hospital.

James was finally convicted for a series of burglaries and ended up in prison for the first time. At an all-time low James has contact with his family, with his partner visiting him with the kids on a regular basis. James regularly self-harmed when he was feeling particularly stressed in prison. James was placed in a shared cell and though having settled into prison life James felt angry and frustrated.

On a recent prison visit his partner told him that the council were planning to change their accommodation because James was no longer living with them. The change in circumstances would mean that James's partner could be moved outside of the local area. James returned to the wing in a low mood. He feels inadequate and powerless to do anything about the change in circumstances. James 'kicks off' in his cell.

Recognising and Identifying Problems

Facilitators encourage the group to use the 7-step model to work through the case study example.

Re-visit case study

James felt daunted by trying out brainstorming. At first he felt that nothing would help the situation. As James and his prison officer started to work together he gained momentum with the situation and provided a number of different ideas which helped him feel more in control of the situation.

'my partner is being forced to move out of her house and is being moved away from the area and I will not see my family every week at visiting time'

Here are some practical solutions

Ask my partner to ring the council and find out where she is moving to

Arrange a specific time when they will be able to visit so I can look forward to the visit

Get some photos of my baby and partner to put up in my cell

Ring my partner more often

Talk to visits to see if I can get extended visiting time when they come

Here are some emotional solutions

Focus on keeping myself to myself and not getting into trouble whilst in prison

Plan the time I have in prison to keep me busy

Ask if I can have extra jobs to do in the prison to keep my mind occupied

Go to the prison gym and take part in some exercise

Consider going to education to see if they have any courses I can attend

Re-visit the case study

When James looked at the initial selection to her brainstorm he decided to cross out. He thought they lacked feasibility and had a negative effect on his wellbeing.

The James grouped the remaining ideas. She grouped them into three categories:

Activities to keep himself busy

Methods of organising time

Strategies to manage stress

After thinking about the advantages and disadvantages of these possibilities James decided that planning time in advance had the advantage of reducing his stress. James solution was first to speak to his personnel officer about contacting education and the jobs section within the prison to find out more about what opportunities were available to him.

James also wanted to ring his partner to discuss the move location and ask her to send some pictures of the baby for his cell wall.

Overall, James thought the combination of these two strategies had a good chance of allowing him to feel better about his time in prison and seeing his family less.

SMART action plan and reviewing progress

SMART – Action plan

**Specific**	clear definition of what the action is
**Measurable**	it can be clearly determined whether or not the action has been achieved
**Achievable**	it is important to break the steps down so they can be successfully completed
**Relevant**	action is linked to the problem
**Time-bound**	timescale by which the action will be achieved

Re-visit the case study

James had to create two action plans one for each solution. They contributed to solving the same problem and could be implemented at the same time.

Extra activities in prison action plan

James decided he would ask his personnel officer about how to approach education and the jobs department in the prison. Once he had done this and gathered some more information James discovered that he needed to complete a jobs request card and place it in the post-box on the wing. He also discovered that he could go to education and speak with one of the teachers directly about what was available. In the next few days James identified the next tasks:

1. He considered his job options and also the potential demand to do such jobs within the prison. He wanted to choose some jobs that he would be allocated quickly, maybe because other people did not want to do them.
2. He decided to speak to his personnel officer again and other prison inmates to try and assess which job options he might have most success with.
3. Once he had gained the information he made a decision to put down a range of job choices with the hope of him being allocated something. These included working in the kitchen and canteen, cleaning on the wings and the toilet areas, working in the workshop making prisoner uniform for other prisons.
4. He was told that he would hear back about his choice of jobs within one week
5. He also planned a visit to education to discuss his course options

Further practice role play examples:

David has been on the wing for 12 months and has become addicted to drugs referred to as SPICE – on the last occasion he only narrowly escaped death and the healthcare staff had to bring him round. They showed him a video of himself and what had happened. He was completely shocked he had no idea how it had affected him and those around him. The video showed him on the floor with the paddles attached. Some members of staff on the video were visibly upset. David decides that this must stop but he doesn't know where to start.

Shane is 17 years old he has been convicted of robbery when he got involved with a gang of youths. He used to live with his grandma and dad who come to visit him regularly. One week his dad comes along to visit him without his grandma to tell him that his grandma has suddenly died from a heart attack. Shane was devastated he did not know how to handle the news – he goes back to his cell and wrecks his pad – and he is taken to the segregation unit to calm down. When he returns to the wing Shane is determined to get himself back on track, he is worried about whether he will be able to attend the funeral and what he can do to support his Dad.

Joseph an older man worked in banking industry for 25 years. He was finally convicted for fraud after a lengthy case and Joseph was sent to prison for 9 years. He was shocked by his incarceration and having been in Leeds for a few months he has been transferred to another prison, but he has struggled to adjust to prison life. He feels completely out of place. He looks like he is not looking after himself – his son comes to visit – he is ashamed of his father's behaviour and tells him he does not want to be part of his life anymore. A member of staff identifies Joseph as needing some help and refers him to you to see how best Joseph can be supported. Joseph feels isolated, alone, and angry, he refuses to come out of his cell when asked to by a member of staff.

Before Patrick came into prison my car was stolen. Patrick was recalled into prison on licence for not following the rules. On the night before he was arrested, he was involved in a car incident which meant that my car was stolen. He doesn't know what has happened to it. He is still paying road tax – He doesn't know if it is still OK or what to do about the insurance. He needs his car on the outside because he is a taxis driver – He is worried that when he is released, he won't be able to work and he won't have any money. He is talking to other prisoners to see if 'one of the boys' on the outside might be able to help, but the problem seems too big he wants to ignore it and pretend it hasn't happened. He thinks he can do nothing about his problem whilst in prison.

Andrew has a long history of self-harm behaviour and he has been monitored by staff for his self-harm behaviour for many months. Andrew has self-harmed himself ever since being in prison. Andrew is quite quiet and generally keeps himself to himself – he has a family and two children outside and he hasn't been able to pay the mortgage on the house for two months. The families' savings have all run out. Last week his wife came to visit in tears saying that she was worried that the house would be re-possessed. This is Andrew's main concern now. He feels completely out of control, he is panicking about what he can do to help support his family and his wife. He doesn't have a clear plan in is head about how he can help to resolve the situation and is feeling very anxious he feels like he needs to advise his wife on what they should do but thinks that time is running out for them. He blames himself for the situation and has been more reluctant to come out of his cell and go to work.
