# Supplementary material for: Cultural adaptation of a UK evidence-based problem-solving intervention to support Polish prisoners at risk of suicidal behaviour: a cross-sectional survey using an Ecological Validity Model
Source: BMJ Open. 2023 Jul 9;13(7):e069252. doi: 10.1136/bmjopen-2022-069252 (PMC10335472; doi:10.1136/bmjopen-2022-069252)
Supplement: Author's manuscript [file bmjopen-2022-069252.draft_revisions.pdf]

BMJ Open is committed to open peer review. As part of this commitment we make the peer review history of every article we publish publicly available.

When an article is published we post the peer reviewers' comments and the authors' responses online. We also post the versions of the paper that were used during peer review. These are the versions that the peer review comments apply to.

The versions of the paper that follow are the versions that were submitted during the peer review process. They are not the versions of record or the final published versions. They should not be cited or distributed as the published version of this manuscript.

BMJ Open is an open access journal and the full, final, typeset and author-corrected version of record of the manuscript is available on our site with no access controls, subscription charges or pay-per-view fees (http://bmjopen.bmj.com).

If you have any questions on BMJ Open's open peer review process please email info.bmjopen@bmj.com

BMJ Open

The international cultural adaptation of a UK problem-solving skills training package: An intervention to address self-harm and suicidal behaviour in custody.

Journal:	*BMJ Open*
Manuscript ID	bmjopen-2022-069252
Article Type:	Original research
Date Submitted by the Author:	16-Nov-2022

Complete List of Authors:	Perry, Amanda; University of York,<br>Zawadzka, Maja; The Academy of Justice<br>Lapinski, Piotr; Józef Piłsudski University of Physical Education in Warsaw<br>Moore, Keeley; Ministry of Justice, HMPS Stocken<br>Rychlik, Jaroslaw; The Academy of Justice<br>Nowak, Beata; The Academy of Justice

Keywords:	MENTAL HEALTH, Suicide & self-harm < PSYCHIATRY, PUBLIC HEALTH

SCHOLARONE™
Manuscripts

I, the Submitting Author has the right to grant and does grant on behalf of all authors of the Work (as defined in the below author licence), an exclusive licence and/or a non-exclusive licence for contributions from authors who are: i) UK Crown employees; ii) where BMJ has agreed a CC-BY licence shall apply, and/or iii) in accordance with the terms applicable for US Federal Government officers or employees acting as part of their official duties; on a worldwide, perpetual, irrevocable, royalty-free basis to BMJ Publishing Group Ltd ("BMJ") its licensees and where the relevant Journal is co-owned by BMJ to the co-owners of the Journal, to publish the Work in this journal and any other BMJ products and to exploit all rights, as set out in our licence.

The Submitting Author accepts and understands that any supply made under these terms is made by BMJ to the Submitting Author unless you are acting as an employee on behalf of your employer or a postgraduate student of an affiliated institution which is paying any applicable article publishing charge ("APC") for Open Access articles. Where the Submitting Author wishes to make the Work available on an Open Access basis (and intends to pay the relevant APC), the terms of reuse of such Open Access shall be governed by a Creative Commons licence – details of these licences and which Creative Commons licence will apply to this Work are set out in our licence referred to above.

Other than as permitted in any relevant BMJ Author's Self Archiving Policies, I confirm this Work has not been accepted for publication elsewhere, is not being considered for publication elsewhere and does not duplicate material already published. I confirm all authors consent to publication of this Work and authorise the granting of this licence.

For peer review only

The international cultural adaptation of a UK problem-solving skills training package: An intervention to address self-harm and suicidal behaviour in custody.

Authors:

Amanda E. Perry*

Maja Zawadzka[1]

Piotr Lapinski[3]

Keeley Moore[2]

Jaroslaw Rychlik[1]

Beata Maria Nowak[1]

(with international collaboration from the group of polish prison officers: Kamila Nowak, Anna Szwedowska, Irmina Chrzanowska, Krzysztof Kasjan, Agnieszka Nowogrodzka, Izabela Stankiewicz, Przemysław Worobiej, Anna Kondraciuk, Bartosz Jaroszewski and Sebastian Lizyńczyk).

*corresponding author

*University of York

Health Sciences Department

2nd Floor ARRC Building

Heslington

York

YO10 5DD

UK

Email: amanda.perry@york.ac.uk

Telephone: 07795315045

1.The University of Justice, Warsaw, Poland.

2.HMPPS: HMP Stocken, Rutland, UK.

3.Central Board of Prisons, Warsaw, Poland

For peer review only

For peer review only - http://bmjopen.bmj.com/site/about/guidelines.xhtml

Cultural adaptation: problem-solving in prisons

KEYWORDS: Cultural, implementation, problem-solving, intervention and prisons

WORD COUNT 3,300.

For peer review only

For peer review only - http://bmjopen.bmj.com/site/about/guidelines.xhtml

STRENGHTS AND LIMITATIONS OF THIS STUDY

An Ecological Validity Model provides a useful structure and aids the cultural adaptation of interventions.

Polish prison staff and students found the intervention to be acceptable and feasible to deliver.

The culturally adapted problem-solving intervention needs further evaluation.

The volunteer purposive nature of the sampling is unlikely to reflect the representative nature of all staff in the Polish prison service.

Use of novel participatory strategies would enhance the representation of this population.

For peer review only

For peer review only - http://bmjopen.bmj.com/site/about/guidelines.xhtml

ABSTRACT

Objectives: This paper documents the adaptation of a UK evidence-based problem-solving intervention to support Polish prison staff and prisoners at risk of suicidal and/or self-harm.

Design, setting and participants: A cross-sectional survey participatory design using an adapted Ecological Validity Model. Collaboration between two male adult prisons, one University and prison officer training centre in Poland. Purposive volunteer participants included, prison officers (n=10), prison staff at two Polish prisons (n=12), staff from the wider prison service (n=39) and students from a Polish University (n=27).

Methods: The adaptation process was underpinned by an adapted Ecological Validity Model. Four stages included: (i) a targeted demonstration for Polish prison staff, (ii) a wider audit of the Polish prison community, (iii) forward and back translation and (iv) two iterative consultations.

Main outcomes: acceptability and feasibility of the training package, reported in a series of bespoke self-report questionnaires using quantitative and qualitative comments.

Results: The evaluation used bespoke questionnaires, descriptive analyses a thematic framework and statistical testing to analyse differences between participant groups.

Conclusions: There was wide acceptance of the skills regardless of participant group indicating that the skills had the potential for widespread appeal across the Polish penitentiary system. The adaptation process produced materials that were deemed to be acceptable and relevant whilst adhering to the comprehensibility of the intervention. Further evaluation of the intervention should be explored using a randomized controlled trial design with Polish prisoners.

For peer review only - http://bmjopen.bmj.com/site/about/guidelines.xhtml

INTRODUCTION

The mental health of people incarcerated in prison is recognized as international public health concern[1 2]. Prisoners experience disproportionate levels of mental health, isolation, boredom, self-harm, and anti-social violent behaviour when compared to the general population [3 2 4 5].

Whilst different practices are used globally to address such health concerns, the international collaborative sharing of best practice between different prison organisations is rarely documented; leading to the potential repetition of work and use of interventions that might not be effective within the unique context of the prison environment [6 7]. Culturally adapting interventions that already have an evidence-base could be an economical and scalable approach that could be considered for further adaptation within different prison systems [8-11].

The prior efficacy of a brief problem-solving therapy (PST) has proven benefit with patients and people in custody who are at risk of self-harm and suicidal behaviour [12 -15]. Individually, randomised trials of PST report positively on outcomes of depression, and allied constructs such as hopelessness [15-17]. Meta-analytical reviews of the evidence utilising data from 21/24 studies showed that PST in comparison to treatment at usual (at final follow-up) was found to reduce symptoms of depression using the Beck Depression Inventory, (MD -3.95, 95% CI -6.05 to -1.86), the Hamilton Depression Rating Scale (MD -0.95, 95% CI -2.54 to 0.65), hopelessness, measured by the Beck Hopelessness Scale (MD -1.38, CI 95% -2.36 to -0.41), suicidal ideation (MD -1.58, CI 95% -1.58 to -0.44) and outcomes of repetition of self-harm at 4 months (OR 0.65, CI 95% 0.36 to 1.16) and final follow-up (OR 0.76, CI 95% 0.55 to 1.05) [18.]

Problem-solving skills have been utilised in the delivery of cognitive behavioural programmes through the UK prison service for many years[19]. Recent endeavours have used co-production methodology to develop a problem-solving training

For peer review only - http://bmjopen.bmj.com/site/about/guidelines.xhtml

package consisting of a workbook, digital materials and a manualised handbook for use with prison staff and delivery of the skills through a peer-mentor scheme [20,18]. Similar to the UK, Poland, has nationwide cognitive-behavioral programs for prisoners (e.g., Aggression Replacement Training: ART)[21-22]. However, in Poland, problem-solving skills are not use with those who present with self-harm behaviour or concerns of mental health and well-being. The following paper therefore uses a four-staged iterative approach to culturally adapt the UK problem-solving model to prevent self-harm and suicidal behaviour for use in Polish prisons.

METHODS

Overall study design

The study was conducted using a collaboration between: The Academy of Justice, in Warsaw, prison staff based at two Polish prisons (ZK Raciborz and ZK Klodzko), trainee students (University of Lodz, Poland) and the University of York, in the UK. An adapted Ecological Validity Model (EVM) [23] included an examination of the use of language, metaphors, and content (i.e., culturally appropriate, and syntonic language), the changing of case study scenarios (relevance and acceptability) and sought to maintain the fundamental therapeutic components of the intervention to retain the intervention comprehensibility and completeness.

The EVM process guided the cultural adaptation using four different stages: (1) a demonstration of the training materials to prison staff and an evaluation of the feasibility and acceptability, (2) a wider audit of the skills to gather feedback from the Polish prison community, (3) adaptation of the current training materials using culturally specific examples, use of forward and back-translation, and (4) two rounds of consultations to identify further amendments to the materials (Figure 1).

[INSERT FIGURE 1 HERE]

For peer review only - http://bmjopen.bmj.com/site/about/guidelines.xhtml

Knowledge user surveys

Three separate surveys were devised by the research team for each phase of the process. Survey one addressed the acceptability of the training and was evaluated using a brief [11-item] self-report questionnaire in November 2019. The questionnaire was aimed at identifying whether the skills were acceptable to Polish prison officers and were perceived as feasible to use within their current job role. The questionnaire elicited a series of yes/no responses and semi-structured qualitative comments.

Survey two was used to inform a wider audit on the use of problem-solving skills within people who were working in the Polish criminal justice system and was distributed during January 2020. Participants were asked to complete a 6-item questionnaire to provide feedback on the acceptability and applicability of the materials and training within their current job roles.

Survey three involved completion of either an 8 or 12-item questionnaire in two rounds of consultation with several staff groups between May 2020 and August 2021.

PROCEDURE

Stage one: Demonstration of the training package for Polish prison staff

The problem-solving demonstration sessions were delivered by KM and AP in The Academy of Justice during a three-day period in November 2019. Ten prison staff members attended the group training by invitation of the Academy to represent different roles across the Polish prison service. The sessions included a demonstration of the problem-solving skills, sharing of the UK case study examples and a series of role play exercises to support adaptation of the materials. At the end of the training session, staff were asked to provide

For peer review only - http://bmjopen.bmj.com/site/about/guidelines.xhtml

feedback using survey one.

Stage two: Audit of the problem-solving skills with staff working in the wider Polish prison community

The purpose of the audit was to gain a wider perspective and greater understanding from a larger population of people who either worked within the prison service or who were undergoing training to become a prison officer or join the army. A purposive volunteer sample of Polish prison staff from the Warsaw Department (39/65 58%) and students studying for a Bachelor in National Security (27/65 42%) at the University of Lodz were invited to take part in the audit. MZ a CBT psychotherapist in Poland with knowledge of the scheme and trained by the PI (AP) demonstrated the problem-support skills with use of a brief animation and teaching of the skills. Following demonstration of the skills staff were invited to complete survey two.

Stage three: Adaptation of the materials into Polish

Feedback from stages one and two informed the translation of the training documents. The translation from English into Polish was completed by MZ. Back translation was completed independently by a translation service in the UK and checked against the original text to identify any differences in meaning or contextual understanding by the project PI (AP). Ratification of the text was completed with further discussion with colleagues in Poland to ensure the wording and language was comparable and culturally appropriate [24].

Stage four: Consultation process of the adapted materials

Two rounds of feedback from staff that had been engaged in stages 1 and 2 and recruitment of a new group of staff (who hadn't seen the materials before) were asked to consult and review the

For peer review only - http://bmjopen.bmj.com/site/about/guidelines.xhtml

training package and feedback using surveys three and four. The findings from the consultation process were categorised into changes relating to the EVM model including language, metaphone and content, relevance and acceptability and intervention comprehensibility and completeness.

Data Analysis

All survey results were anonymized during digital input into a series of different Excel spreadsheets. IBM SPSS Statistics (version 26). The quantitative responses were used to generate mean scores and the qualitative comments were summarized at each stage in the study using a thematic framework [25]. Qualitative comments within each theme were additionally 'counted' to provide a numerical frequency which enabled a direct comparison of comments within each theme. The responses from stage 2 were divided into those reporting either by prison officers (PO) or students (S) in training.

Statistical assumptions were used to identify use of parametric or nonparametric testing at ($p<0.05$). Statistical analyses were conducted using Statistica StatSOFT version 13. Specific hypotheses were generated a priori for stage 2. It was hypothesized that the materials would be more acceptable to those already working in the prison environment as opposed to those students who were in training; because they might have through experience, a better understanding of the application of the skills.

PATIENT AND PUBLIC INVOLVEMENT

Patients and public were involved throughout the consultation process. Patients and public feed into the adaptation of the training package and as a result changes were made based on their feedback.

For peer review only - http://bmjopen.bmj.com/site/about/guidelines.xhtml

RESULTS

Stage one: Demonstration of the training package for Polish prison staff

Characteristics of the training sample

Prison staff (n=10) were aged between 29 and 39 years of age (mean age 34.7 years, SD = 3.25). All identified as white European and spoke Polish as their first language. Most of the group were female 7/10 (70%), and one participant considered themselves disabled. Participants had worked in the prison service for an average of 103.4 months (SD = 39.8, min = 51 months, max = 178 months), and within the current prison for a mean number of 65.7 months (SD = 35.5, min = 6, max = 111). All staff reported dealing with an incident of self-harm or attempted suicide within an average of 16 months prior (SD = 15.8, min = 1 month, max = 36 months). Of those reported incidents 1/10 (10%), reported self-poisoning, 3/10 (30%) reported general self-injury four reported incidence of attempted suicide 4/10 (40%). Incidence of violence was reported by 7/10 (70%) of staff reported an experience of being involved in a violent incident just over a third of staff reported that this could have been avoided 3/10 (30%). Most prison staff 7/10 (70%) encountered prisoners with mental health problems, just under a quarter experienced these on a daily or weekly basis 2/10 (20%).

Acceptability of the training package and use of the skills

All participants thought that the training was useful, and most learned new skills. Half the group thought the training should have been longer and nearly all felt that a booster session would have been useful (Table 1). Some of the main themes included: an opportunity to learn new skills (‘new knowledge’ and a ‘different approach is always useful’), providing useful communication skills (‘learning how to speak to prisoners’), developing reflection of perspective (‘to view it through the

For peer review only - http://bmjopen.bmj.com/site/about/guidelines.xhtml

prisoner's perspective'), use of a collaborative approach ('working towards a joint search for a solution, not imposing one views'), and use of open-ended questions to progress behaviour change ('work on changes in thinking is important'). Most staff felt that training could be delivered by prisoners, prison staff, psychologists, educators, and practitioners within the prison site. Further adaptation of the materials and example case studies were welcomed to expand on the existing training opportunity; half the group wanted the training to be longer and nearly all thought that an additional booster session would be useful.

Table 1: Results following demonstration of the training package for Polish prison staff

Questionnaire	n/10 (% yes)	Qualitative comments
In what ways was the training helpful to you?	NA	Development of a new perspective<br>A new technique/tool kit/ alternative approach<br>Exchange of ideas between the UK & Poland
What are the positive aspects of the scheme?	NA	Straight forward, simple, easy to understand<br>Problems solved by prisoners<br>The development of coping skills<br>Group work<br>Providing a structure to help change
Is the training applicable for your job role?	10/10 (100%)	NA
Did you learn any new skills?	7/10 (70%)	NA
If yes, what new skills did you learn?	NA	Working from the prisoners perspective<br>How to speak to prisoners<br>Development of problem-solving skills
Who do you think is best to offer the training to prison staff or prisoners?	NA	Prisoners, prison staff, psychologists, educators, practitioners within the prison site.

For peer review only - http://bmjopen.bmj.com/site/about/guidelines.xhtml

Is the training relevant to your job role in the prison?	9/10 (90%)	NA
What improvements could be made to the scheme?	NA	Development of more practice exercises<br>Use of additional case study examples<br>Development of the materials
Is the length of the training adequate?	5/10 (50%)	The training should be longer.
Would it be useful to have a booster session?	9/10 (90%)	NA
How will the training be sustained?	NA	In everyday use<br>Training of prisoners on an individual basis<br>Training as part of prisoner officer rules

Stage two: Audit of the problem-solving skills with staff working in the wider Polish prison community

Characteristics of the audit sample

A volunteer purposive sample of 67 persons took part in the wider audit: 39/67 (58%) were prison staff and 28/67 (42%) were students. Of the prison staff 30/39 (77%) identified as male. The age ranged from 20 to 50 years of age; 12/39 (31%) worked as educators. Other staff (3/39, 7%) worked in prison management, 15/39 (38%) in the Security Department, 10/39 (25%) as a shift commander; 1/39 (2%) in the Finance Department, 2/39 (5%) in the Quartermaster Department, 3/39 (7.8%) in the Therapeutic Department; and 2/39 (5%) persons defined themselves as an Inspector. Students were aged between 20 and 30 years of age: 9/28 (32%) were male from the University of Lodz. All students were working towards a Masters degree in National Security with an aim to join the prison service or army.

For peer review only - http://bmjopen.bmj.com/site/about/guidelines.xhtml

For peer review only

Overall results from the audit

Nearly all prison staff and students thought that problem-solving skills should be taught as part of standard training, over (53/67 80%) (regardless of whether they were students or prison staff) thought that there were benefits to using the skills (Table 2). The perceived benefits were wide ranging. Most reported by prison officers was the perceived opportunity to change behaviour and the ability to empower others to increase the pace of decision making (28/67 42%) and develop knowledge (30/67 45%) and apply the skills in instances of crisis management (18/67 27%). Students were most likely to report use of the skills in general problem solving (22/67 33%). Staff were more likely that students (37% vs 11%) to use the skills for self-development and knowledge. Students were more likely than staff (35% vs 15%) to use the skills to develop their own coping strategies. Over half of prison staff (23/39 58%) and most students (19/28 70%) saw relevance of the skills within their job role.

No significant difference were found in the opinion of students or staff in the perceived beneficial use of the training being incorporated into the already established training scheme for newly qualified prison officers ($X^2(1, N = 66) = 3.45$, p > 05.). Prison officers when compared to students were significantly more likely to accept that the training could be translated into skills for prisoners **(**U=51, median rank: 243; 222), p<0.05.

Table 2: Audit of the acceptability of the problem-solving skills with a wider Polish prison community

Questionnaire items	PO (N% yes)	Student (N% yes)	Prison Officer (PO) and Student (S) Qualitative themes	
Do you think problem-solving skills should be taught to	38/39 (97%)	23/27 (85%)	NA	NA

For peer review only

prison officers as part of standard training?				
Do you think there are benefits from learning the skills?	34/39 (87%)	24/27 (88%)	NA	NA
If yes, what are the benefits?	NA	NA	Changing behaviour (PO=9/33 27% vs S=2/24 8%)<br>Crisis planning & prevention (PO=2/33 6% vs S=3/24 12%)<br>Developing coping strategies (PO=2/33 6% vs S=3/24 12%)<br>Developing personal skills (PO=1/33 3% vs S=1/24 4%)<br>Developing social networks (PO=4/33 12% vs S=1/24 4%)<br>Generating solutions (PO=1/33 3% vs S 1/24 4%)<br>Helping someone (PO=0/33 0% vs 5/24 20%)<br>General problem solving (PO=2/33 6% vs S=8/24 33%)<br>Empowerment to make decisions at pace (PO=14/33 42% vs 2/24 8%).	
What do you like most about the idea of using the skills?	NA	NA	Ability to compromise (PO=3/32 9% vs S=0/17 0%)<br>Knowledge & self-development (PO=12/32 37% vs S=2/17 11%)<br>Developing coping strategies (PO=5/32 15% vs S=6/17 35%)<br>Empowerment (PO=6/32 18% vs S=5/17 29%)<br>Tailored to individual need (PO=1/32 3% vs S=0/17 0%)<br>Improved communication (PO=1/32 3% vs S=2/17 11%)<br>Accessible to help others (PO=4/32 12% vs S=2/17 11%)	
Could you see them being used in your job role?	23/39 (58%)	19/27 (70%)	NA	NA
If yes, in what capacity?	NA	NA	Solving problems (PO=1/22 4% vs S=3/16 18%)<br>Helping others (PO=0/22 0% vs S=3/16 18%)<br>Applicability in life experiences (PO=0/22 0% vs S=2/16 12%)<br>Developing empathy (PO=0/22 0% vs S=2/16 12%)<br>Crisis management (PO=6/22 27% vs S=4/16 25%)	

For peer review only - http://bmjopen.bmj.com/site/about/guidelines.xhtml

			Increased knowledge (PO=10/22 45% vs S=1/16 6%)<br>Empowerment (PO=3/22 13% vs S=1/16 6%)<br>Social cultural activities (PO=2/22 4% vs S=0/16 0%)

Stage three: Adaptation of the materials into Polish

Feedback from stages 1 and 2 allowed the materials to be translated into Polish using a series of changes within the structure of the EVM. These included:

Language, metaphors, content

Words were added to the workbooks (e.g., 'to be' or 'to have') to make the context more understandable in Polish. Words were also changed to reflect different cultural differences. In addition, some English words were non-existent in Polish and in these cases similar words were found and replaced to ensure that the comprehension of the materials remained the same.

Relevance and acceptability

One male case study was reviewed as part of the training package (Appendix 1). Changes to the case study ensured it's applicability to the Polish penitentiary system. Changes to the context and background information of the case study to reflect the daily routine of prisoners in Poland. Sources of support were changed to reflect the provision of support within the context of the Polish prison system. Options generated from the case study that did not exist within the Polish context were changed to reflect forms of assistance available in Poland.

Intervention comprehensibility and completeness

The workbook titles were changed to reflect the comments made during stages 1 and 2. The theoretical underpinning of the intervention was adhered to throughout the

For peer review only

structure of the workbooks to ensure that the intervention comprehensibility was maintained. British names of resources were changed to Polish resources to enable the completeness of the intervention to be supported.

Stage four: Consultation process of the adapted materials

Characteristics of the sample: first and second consultations

Consultation of the adapted materials included two rounds. In round one participants included (i) prison staff from stage one (8/10 80%), and a (ii) wider group of professionals (10/39 25%) from stage two. Round two included those participants who took part in round one plus a group of serving prisoner officers (n=12) from the prisons of ZK Racibórz and ZK Kłodzko who had not previously seen the materials. (Table 3).

Table 3: Consultations process of adapted materials round two

Second consultation (n=28)	Definitely not (yes)	Rather not (yes)	No strong preference	Rather (yes)	Definitely (yes)
Quality	0	1 (4%)	2 (7%)	15 (53%)	10 (36%)
Clarity	0	0	5 (18%)	12 (43%)	11 (39%)
Diagrams	0	1 (4%)	2 (7%)	13 (46%)	13 (46%)
Structure	0	2 (7%)	2 (7%)	15 53%)	8 (29%)
Materials	0	0	4 (14%)	17 (60%)	6 (21%)
Training modules	0	2 (7%)	2 (7%)	14 (50%)	9 (32%)
Understanding clarity and content	0	1(4%)	5 (18%)	14 (50%)	7 (25%)
Acceptability of the case study	1 (4%)	1 (4%)	2 (7%)	15 53%)	8 (29%)
Language adaptation	1 (4%)	1 (4%)	2 (7%)	14 (50%)	10 (36%)
Possibility of self-development	1 (4%)	1 (4%)	5 (18%)	18 (64%)	3 (11%)

For peer review only - http://bmjopen.bmj.com/site/about/guidelines.xhtml

For peer review only

Development of coping strategies	0	2 (7%)	3 (11%)	14 (50%)	8 (29%)
Level of innovation: use within the system	0	1 (4%)	3 (11%)	14 (50%)	9 (32%)

Language, metaphors, content

The combined results from consultations one and two showed that overall, the language used were appropriate for the prison context ('I rate the materials at a level appropriate to the subjects' language. 'I think it is understandable and also simple'). The second consultation process did identify some useful further iteration on use of appropriate language to ('unify terminology' and reflected that 'some words are not good in non-changed from original version form'). On the basis of this feedback a final iteration of the materials was completed.

Relevance and acceptability

The workbooks contained diagrams and pictorial representations through cartoon like characters. These were used in the original UK workbooks to aid literacy levels[26 27]. In Poland, most prison staff thought these were acceptable with wider benefit to other client groups recognized ('the presented training materials are interesting their great value is universalism'; they can be implemented with different groups of people'). The general opinions about the overall presentation of the workbooks were good ('diagram and pictures facilitate understanding of the presented content'). Further amendments were made to the case study to reflect more closely the structure of families in Poland.

DISCUSSION

To our knowledge this study is the first to demonstrate use of the EVM to develop the cultural adaptation of an evidenced-based UK problem solving skills training

For peer review only - http://bmjopen.bmj.com/site/about/guidelines.xhtml

package for prison staff and prisoners at risk of self-harm, mental health and well-being for use in Poland. Cultural adaptation procedures are key in the acceptability and recognized feasibility of using any intervention [6]. Iterative input from Polish prison staff was vital in achieving a product that has the potential to support good adherence with a group of people that are inherently regarded as difficult to engage. Poor adherence to interventions is a known primary barrier to achieving optimal treatment outcomes for mental health worldwide [28].

The results demonstrated wide acceptance of the skills regardless of staff or student group, indicating that the use of the skills had potential widespread appeal across the Polish penitentiary system. As expected, staff in comparison to students could envisage greater application of the skills within the prison context. This may well reflect their experience of working within the prison environment. The guiding principle of cultural adaptation is to work closely with stakeholders during early developmental stages to ensure that they can be sustained [29-30]. Prisons are challenging settings, trust, confidentiality, and fairness are more difficult to establish in prison settings [31]. Failure to acknowledge something as acceptable or feasible is a barrier to implementation and wide-spread use. Interventions that engage staff from across the prison sites help consider the integration and development of sustainable models to prevent self-harm behaviour and promote mental health and well-being going forward [3].

Implications and future research

Our adaptation processes resulted in good levels of acceptability and feasibility, with positive feedback from prison staff and students. Recommendations refer to the necessity of using problem-solving skills as a part of standard training, by integrating skills such as role-play to support communication skills. Further development of the workbook and training package need to consider adaptations to ensure that the materials are widely accessible to prisoners with different protected

For peer review only - http://bmjopen.bmj.com/site/about/guidelines.xhtml

characteristics. The inclusion of further narrative case studies for females in prison would require additional consideration. Further research is required to test the effectiveness of the intervention package using a randomized controlled trial design.

Limitations

The study has several limitations. First, the volunteer purposive nature of the sampling is unlikely to reflect the representative nature of all staff in the Polish prison service. Arguably, the nature of the sampling within this evaluation may impact on the generalisability of the training package. Future extension of this work would require feedback from a diverse range of prisoners. Use of novel participatory strategies would enhance the representation of this population. An additional multidisciplinary independent steering group would be recommended to expand any subsequent consultation process.

CONCLUSION

The process of cultural adaptation enables researchers to efficiently tailor evidence-informed interventions for diverse populations while retaining the core components that underpin efficacy. The EVM provides a useful framework for adapting interventions. Use of this method produced an acceptable training package for the Polish prison service. Further evaluation of the effectiveness of the problem-solving training skills needs to be explored using a randomised controlled trial design.

DATA AVAILABILITY STATEMENT

Data is made available upon reasonable request

ETHICS STATEMENTS

For peer review only - http://bmjopen.bmj.com/site/about/guidelines.xhtml

Ethical approval for the study was granted by the Department of Health Sciences at the University of York in the UK and reviewed by a RODO Data Protection Specialist in The Academy of Justice, in Warsaw, Poland. Permission was granted from each prison in taking part in the study. All study participants received full informed consent.

ACKNOWLEDGEMENTS

With many thanks to the UK HMPPS Intervention services, to all the prison staff that contributed to the project including the prisons of ZK Racibórz and ZK Kłodzko. To the Central Board of Prisons in Poland who gave permission for this research.

FUNDING

This study was funded through the Wellcome Trust, Centre for Future Health, Rapid Response Fund, University .of York, UK. No grant number provided.

AUTHOR CONTRIBUTION STATEMENT

AP/MZ devised the study with the initial support from PL. AP and KM delivered the training to the prison officers in Poland. JR conducted the analysis and BNM supported Polish colleagues in the written work for the publication. All elements of the project were overseen by AP. All authors worked on the draft of the manuscript.

FIGURE LEGEND

Figure 1: Study flow process

COMPETING INTEREST STATEMENT

No competing interest

For peer review only - http://bmjopen.bmj.com/site/about/guidelines.xhtml

For peer review only

Cultural adaptation: problem-solving in prisons

ORCID: Amanda E Perry: http://orcid.org/0000-0002-0279-1884

For peer review only

For peer review only - http://bmjopen.bmj.com/site/about/guidelines.xhtml

REFERENCES

1. Durcan, G. (2021). The future of prison mental health care in England. M Centre for mental health. [CentreforMentalHealth_TheFutureofPrisonMentalHealthCare_0.pdf]. Accessed September 2022.

2. Fazel, S., Hayes, A. J., Bartellas, K., Clerici, M., & Trestman, R. (2016). Mental health of prisoners: prevalence, adverse outcomes, and interventions. Lancet Psychiatry, 3, 871-881 doi: 810.1016/S2215-0366(1016)30142-30140. Epub 32016 Jul 30114. PMID: 27426440; PMCID: PMC25008459

3. Favril, L., Yu, R., Hawton, K., & Fazel, S. (2020). Risk factors for self-harm in prison: a systematic review and meta-analysis of 35 studies covering 663735 adults. Lancet Psychiatry, 7(8), 682-691. doi: 610.1016/S2215-0366(1020)30190-30195.

4. Brugha, T., Singleton N, Meltzer H, Bebbington P, Farrell M, & Jenkins R, G., L. (2005). Psychosis in the community and in prisons: a report from the British National Survey of psychiatric morbidity. American Journal of Psychiatry, Apr;162(4):774-80. doi: 10.1176/appi.ajp.162.4.774. PMID: 15800152. 774-780.

5. Favril, L., Indig, D., Gear, C. *et al.* (2020) Mental disorders and risk of suicide attempt in prisoners. *Soc Psychiatry Psychiatr Epidemiol* **55**, 1145–1155. https://doi.org/10.1007/s00127-020-01851.

6. Culbert, G.J. & Williams, A. B. (2018). Cultural Adaptation of a Medication Adherence Intervention With Prisoners Living With HIV in Indonesia: A Pragmatic Approach to Intervention Development. Journal of the Association of Nurses in AIDS Care, 29, Issue 3, Pages 454-465,ISSN 1055-3290, https://doi.org/10.1016/j.jana.2017.11.006.

7. Thakker, J. (2014). Cultural factors in offender treatment: Current approaches in New Zealand. Social and Behavioural Sciences, 113, 213-223. DO 10.1016/j.sbspro.2014.01.028.

For peer review only - http://bmjopen.bmj.com/site/about/guidelines.xhtml

8. Castro, F. G., Barrera, M. J., & Holleran Steiker, L. K. (2010). Issues and challenges in the design of culturally adapted evidence-based interventions. Annual Rev Clin Psychology 6:213-39. doi: 10.1146/annurev-clinpsy-033109-132032.

9. Faregh, N., Lencucha, R., Ventevogel, P., Dubale, B. W., & Kirmayer, L. J. (2019). Considering culture, context and community in mhGAP implementation and training: challenges and recommendations from the field. Int J Ment Health Syst 13:58. doi: 10.1186/s13033-019-0312-9.

10. Li W, Zhang L, Luo X, Liu B, Liu Z, & Lin F, e. a. (2017). A qualitative study to explore views of patients', carers' and mental health professionals' to inform cultural adaptation of CBT for psychosis (CBTp) in China. BMC Psychiatry, 17(1):131. doi: 10.1186/s12888-017-1290-6.

11. Reingle Gonzalez, J. M., & Connell, N. M. (2014). Mental health of prisoners: identifying barriers to mental health treatment and medication continuity. American journal of public health, 104(12), 2328-2333. https://doi.org/10.2105/AJPH.2014.302043.

12. Demiris, G., Oliver, D., Washington, K., Fruehling, L. T., Haggarty-Robbins, D., Doorenbos, A., Berry, D. (2010). A Problem-Solving Intervention for Hospice Care Givers: a pilot study. Journal of Palliative Medicine, 13(8), 1005-1011 https://doi.org/1010.1089/jpm.2010.0022.

13. McLeavey, B. C., Daly, R. J., Ludgate, J. W., & Murray, C. M. (1994). Interpersonal problem-solving skills training in the treatment of self-poisoning patients. Suicide Life Threat Behav, 24(4), 382-394. doi:10.1111/j.1943-278X.1994.tb00817.x

14. Mehlum L, Tørmoen AJ, Ramberg M, Haga E, Diep LM, Laberg S, Larsson BS, Stanley BH, Miller AL, Sund AM, Grøholt B (2014). Dialectical behavior therapy for adolescents with repeated suicidal and self-harming behavior: a randomized trial. J Am Acad Child Adolesc Psychiatry.Oct;53(10):1082-91. doi: 10.1016/j.jaac.2014.07.003. Epub 2014 Jul 22. PMID: 25245352.

For peer review only - http://bmjopen.bmj.com/site/about/guidelines.xhtml

15. Xavier, A., Otero, P., Blanco, V., & Vazquez, F. L. (2019). Efficacy of a problem-solving intervention for the indicated prevention of suicidal risk in young Brazilians: Randomized controlled trial. Suicide and Life Threatening Behaviour, 49, 1746-1761 https://doi.org/10.1111/sltb.12568.

16. Breuer, E., Lee, L., & De Silva, M. (2016). Using theory of change to design and evaluate public health interventions: a systematic review. Implementation Science, 11, 63 https://doi.org/10.1186/s13012-016-0422-6.

17. World Health Organisation. (2019). Status report on prison health in the WHO European Region. Copenhagen: WHO Regional Office for Europe. https://apps.who.int/iris/handle/10665/329943 [Accessed September 2022].

18. Perry, A. E., Waterman, M., Dale, V., Moore, K., & House, A. (2021). The effect of a problem support intervention on self-harm and violence in prison: an interrupted time series analysis using routinely collected prison data. EClinical Medicine- The Lancet, 32 (1007021), 1-11 https://doi.org/10.1016/j.eclinm.2020.100702.

19. Gobbett MJ, & Sellen, J.L. (2014). An evaluation of the HM prison service "thinking skills programme" using psychometric assessments. Int J Offender Therapy Comp Criminol., Apr;58(4):454-73. doi: 10.1177/0306624X12472485. Epub 2013 Feb 6. PMID: 23390065.

20. Perry A. E., Waterman MG, House A*, et al* Problem-solving training: assessing the feasibility and acceptability of delivering and evaluating a problem-solving training model for front-line prison staff and prisoners who self-harm *BMJ Open* 2019;**9:**e026095. doi: 10.1136/bmjopen-2018-026095.

For peer review only - http://bmjopen.bmj.com/site/about/guidelines.xhtml

21. Majcherczyk, A. (2011). Selected rehabilitation programs - theoretical and practical perspective. *Przegląd Więziennictwa Polskiego", nr 70, p. 5-30.*
22. Rejzner, A., & Szczepaniak, P. (2009). Therapy in rehabilitation, volume I Theoretical approach & volume II A practical approach. Warszawa: Żak.
23. Bernal, G., Bonilla, J., & Bellido, C. (1995). Ecological validity and cultural sensitivity for outcome research: issues for the cultural adaptation and development of psychosocial treatments with Hispanics. J Abnorm Child Psychol 23(1):67-82. doi: 10.1007/BF01447045.
24. Ozolins, U., Hale, S., Cheng, S. X., Hyatt, A., & Schofield, P. T. (2020). Translation and back-translation methodology in health research - a critique. Expert Review of Pharmacoeconomics & Outcomes Research, DO 10.1080/14737167.2020.1734453.
25. Braun, V., & Clarke, V. (2013). Successful qualitative research: A practical guide for beginners. London: Sage.
26. Coates, S. (2016). Unlocking Potential: A review of education in prison. Ministry of Justice. https://www.gov.uk/government/publications/unlocking-potential-a-review-of-education-in-prison. [Accessed September 2022].
27. Morken, F., Jones, L. Ø., & Helland, W. A. (2021). Disorders of Language and Literacy in the Prison Population:A Scoping Review. Educ. Sci., 11, 77. https://doi.org/10.3390/ educsci11020077. Najwyższa, I. K. (2020). Security of inmates. Information on the inspection results. KPB.430.002.2020, Nr ewid. 52/2020/P/19/040/KPB, available online: https://www.nik.gov.pl/plik/id,22869,vp,25572.pdf [Accessed January 2022].

For peer review only - http://bmjopen.bmj.com/site/about/guidelines.xhtml

28. DiMatteo, M. R., Giordani, P. J., Lepper, H. S., & Croghan, T. W. (2002). Patient Adherence and Medical Treatment Outcomes A Meta-Analysis. *Medical Care*, *40* (9), 794–811. http://www.jstor.org/stable/3768145.

29. Castro, F. G., & Yasui, M. (2017). Advances in EBI development for diverse populations: towards a science of intervention adaptation. Prevention Science, 1-7. doi: 10.1007/s11121-017-0809-x.

30. Williams, A. B., Wang, H., Burgess, J., & X., L. (2013). Cultural adaptation of an evidence-based nursing intervention to improve medication adherence among people living with HIV/AIDS (PLWHA) in China. International Journal of Nursing Studies, 50(4):487-494. doi: 10.1016/j.ijnurstu.2012.08.018.

31. Azbel, L., Grishaev, Y., Wickersham, J. A., Chernova, O., Dvoryak, S., Polonsky, M., & Altice, F. L. (2016). Trials and tribulations of conducting bio-behavioral surveys in prisons: implementation science and lessons from Ukraine. International Journal of Prisoner Health, 12(2):78-87. doi: 10.1108/IJPH-10-2014-0041. 25245352.

For peer review only

For peer review only - http://bmjopen.bmj.com/site/about/guidelines.xhtml

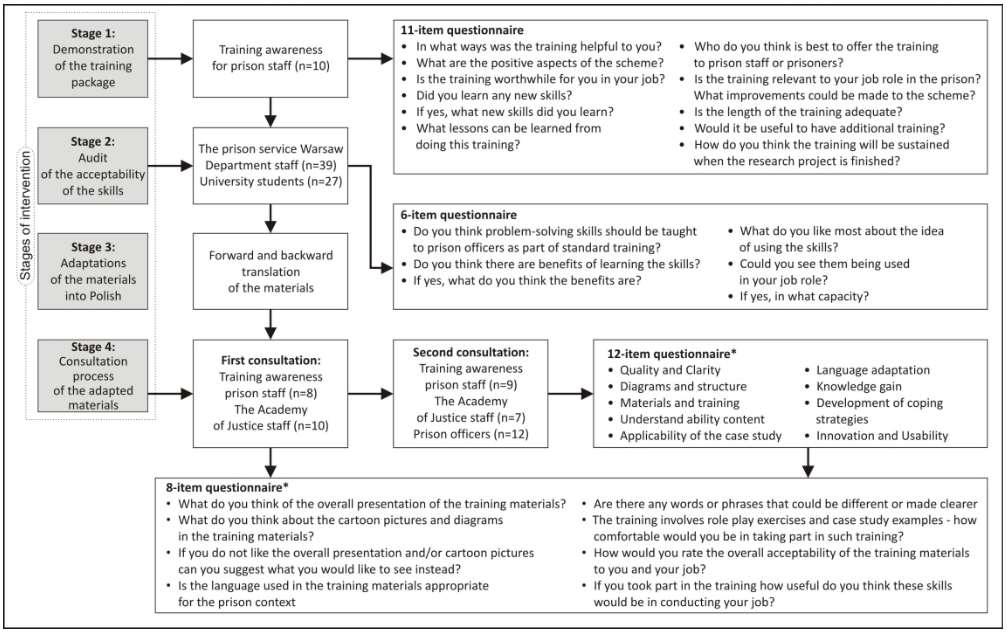

Figure 1: Study Flow Process

263x164mm (300 x 300 DPI)

Appendix 1

Male Case Study Example:

James is 22 years old. He is one of six siblings and lives with his father and stepmother. James's relationship with his parents was troubled from a young age and his father would come home drunk and beat James. His stepmother found it difficult to deal with James's aggressive emotional outbursts and he was excluded from school at age 11 for poor behaviour and angry outbursts.

James started to mix with a gang of older boys who were known in the area for committing petty crimes. He became involved in drugs at age 13 years and was caught by the police for burglary when he was 16 years old. He also had a series of relationships with older women which led to a number of pregnancies resulting in two sons and a new baby. James's stepmother was unable to control his behaviour and did not want him in the house anymore so James was asked to leave.

James went to stay with a friend but soon ended up living in a hostel. He found it difficult to get a job and ended up stealing to support his drug habit. His physical and mental health deteriorated and he no longer took care of himself. One day he took drugs and alcohol and ended up in the Accident and Emergency department at the hospital.

James was finally convicted for a series of burglaries and ended up in prison for the first time. At an all-time low James has contact with his family, with his partner visiting him with the kids on a regular basis. James regularly self-harmed when he was feeling particularly stressed in prison. James was placed in a shared cell and though having settled into prison life James felt angry and frustrated.

On a recent prison visit his partner told him that the council were planning to change their accommodation because James was no longer living with them. The change in circumstances would mean that James's partner could be moved outside of the local area. James returned to the wing in a low mood. He feels inadequate and powerless to do anything about the change in circumstances. James 'kicks off' in his cell.

For peer review only

BMJ Open

Use of an Ecological Validity Model to address the international cultural adaptation of a UK problem-solving skills training package: An intervention to address self-harm and suicidal behaviour for people in custody in Polish prisons.

Journal:	*BMJ Open*
Manuscript ID	bmjopen-2022-069252.R1
Article Type:	Original research
Date Submitted by the Author:	11-May-2023
Complete List of Authors:	Perry, Amanda; University of York,<br>Zawadzka, Maja; The Academy of Justice<br>Lapinski, Piotr; University of Physical Education in Warsaw, Faculty of Rehabilitation, Department of Occupational Therapy.<br>Moore, Keeley; Ministry of Justice, HMPS Stocken<br>Rychlik, Jaroslaw; The Academy of Justice<br>Nowak, Beata; The Academy of Justice
**Primary Subject Heading**:	Mental health
Secondary Subject Heading:	Evidence based practice, Global health, Legal and forensic medicine, Patient-centred medicine
Keywords:	MENTAL HEALTH, Suicide & self-harm < PSYCHIATRY, PUBLIC HEALTH

SCHOLARONE™
Manuscripts

I, the Submitting Author has the right to grant and does grant on behalf of all authors of the Work (as defined in the below author licence), an exclusive licence and/or a non-exclusive licence for contributions from authors who are: i) UK Crown employees; ii) where BMJ has agreed a CC-BY licence shall apply, and/or iii) in accordance with the terms applicable for US Federal Government officers or employees acting as part of their official duties; on a worldwide, perpetual, irrevocable, royalty-free basis to BMJ Publishing Group Ltd ("BMJ") its licensees and where the relevant Journal is co-owned by BMJ to the co-owners of the Journal, to publish the Work in this journal and any other BMJ products and to exploit all rights, as set out in our licence.

The Submitting Author accepts and understands that any supply made under these terms is made by BMJ to the Submitting Author unless you are acting as an employee on behalf of your employer or a postgraduate student of an affiliated institution which is paying any applicable article publishing charge ("APC") for Open Access articles. Where the Submitting Author wishes to make the Work available on an Open Access basis (and intends to pay the relevant APC), the terms of reuse of such Open Access shall be governed by a Creative Commons licence – details of these licences and which Creative Commons licence will apply to this Work are set out in our licence referred to above.

Other than as permitted in any relevant BMJ Author's Self Archiving Policies, I confirm this Work has not been accepted for publication elsewhere, is not being considered for publication elsewhere and does not duplicate material already published. I confirm all authors consent to publication of this Work and authorise the granting of this licence.

For peer review only

Use of an Ecological Validity Model to address the international cultural adaptation of a UK problem-solving skills training package: An intervention to address self-harm and suicidal behaviour for people in custody in Polish prisons.

Authors:
Amanda E. Perry*
Maja Zawadzka[1]
Piotr Lapinski[3]
Keeley Moore[2]
Jaroslaw Rychlik[1]
Beata Maria Nowak[1]

(with international collaboration from the group of polish prison officers: Kamila Nowak, Anna Szwedowska, Irmina Chrzanowska, Krzysztof Kasjan, Agnieszka Nowogrodzka, Izabela Stankiewicz, Przemysław Worobiej, Anna Kondraciuk, Bartosz Jaroszewski and Sebastian Lizyńczyk).

*corresponding author

*University of York
Health Sciences Department
2nd Floor ARRC Building
Heslington
York
YO10 5DD
UK
Email: amanda.perry@york.ac.uk
Telephone:+44(0)7795315045

1.The Academy of Justice, Warsaw, Poland.

2.HMPPS: HMP Stocken, Rutland, UK.

3. University of Physical Education in Warsaw, Faculty of Rehabilitation, Department of Occupational Therapy.

KEYWORDS: Cultural, implementation, problem-solving, suicidal and prisons

WORD COUNT 4173

For peer review only - http://bmjopen.bmj.com/site/about/guidelines.xhtml

Cultural adaptation: problem-solving in prisons

ABSTRACT

Objective: To complete a cultural adaptation of a UK evidence-based problem-solving intervention to support Polish prisoners at risk of suicidal behaviour.

Design: A cross-sectional survey participatory design using an Ecological Validity Model.

Setting: The study was a collaboration between: The Academy of Justice, in Warsaw, the University of Lodz, two Polish prisons (ZK Raciborz and ZK Klodzko), and the University of York (UK).

Methods: The adaptation process included an examination of the use of language, metaphors, and content (i.e., culturally appropriate, and syntonic language), the changing of case study scenarios (relevance and acceptability) and maintenance of the intervention comprehensibility and completeness. Four stages included: (i) a targeted demonstration for Polish prison staff, (ii) a wider audit of the perceived benefit of such skills with Polish prison staff, (iii) forward and back translation of the adapted package and (iv) two iterative consultations with participants from stages (i) and (ii) and prison officers.

Participants: Self-selecting volunteer participants included, targeted prison staff (n=10), prison staff from the wider Polish prison service (n=39), students from the University of Lodz (n=28), and prison officers from two Polish prisons (n=12).

Main outcomes and measures: Acceptability and feasibility of the training package, reported in a series of knowledge user surveys.

Results: Recognised benefits of using the training package included: enhancing communication, reflective development, collaborative working, changing behaviour, empowering decision making, use in crisis management situations and use of open-ended questions. The skills were endorsed to be used as part of future penitentiary training for prison officers.

Conclusions: The skills had widespread appeal for use across the Polish penitentiary system. The materials were deemed relevant whilst adhering to the comprehensibility of the intervention. Further evaluation of the intervention should be explored using a randomized controlled trial design.

For peer review only - http://bmjopen.bmj.com/site/about/guidelines.xhtml

STRENGTHS AND LIMITATIONS OF THIS STUDY

- *Cultural adaptations of prison interventions are rare.*
- *An Ecological Validity Model was used to adapt a UK problem-solving intervention for use within Polish prisons.*
- *The study used small volunteer self-selecting groups of prison staff, prison officers and students who were training to be prison officers.*
- *Future engagement with prisoners is key to understanding more about the acceptability and feasibility of the intervention delivery.*
- *A randomised controlled trial is needed to measure the effectiveness of the adapted problem-solving intervention.*

INTRODUCTION

The mental health of people incarcerated in prison is recognized as an international public health concern[1,2]. Prisoners experience disproportionate levels of mental health, isolation, boredom, self-harm, and anti-social violent behaviour when compared to the general population [2-5]. Prisoners are more than five times more likely to suffer from a common mental health disorder (such as depression) and once diagnosed are more likely to reoffend [6,7] with demand often outstripping available resources[8]. In the last five years, UK and European prisons have reported rises in the incidence of violence assaults and self-harm[9]. Interpretation of this data is, in part also about understanding some of the cultural differences in practices and policy.

Poland and the UK have similar prison populations and official statistics of the Polish prison system show rates of suicide, self-harm and aggressive behaviour (https://sw.gov.pl/strona/Statystyka). Although, rates of suicide and self-harm behaviour in Poland are relatively low; (between 2015-2019 representing 2.53-3.38 per 10,000 prisoners)[10], cultural differences between Poland and the UK reflect different policies for recording of self-harm behaviour. In Poland, changes to the legal provision of recording self-harm in 2013 was restricted to the inclusion of only those incidents where cause to harm lasted **more** than seven days. Current self-harm rates recorded in Poland are therefore likely to be significantly less than the actual number of incidents. In addition, stigma associated with reporting mental health problems in Poland has

For peer review only - http://bmjopen.bmj.com/site/about/guidelines.xhtml

been noted as a barrier to prisoners raising concerns about mental ill health[11].

Despite the international recognition of the disproportionate rates of suicide and self-harm behaviour in prisons, there is little evidence to demonstrate the sharing of best practice. Often, interventions devised from community populations are therefore not relevant, without further adaptation to the prison environment[12,13,14,15]. This is particularly relevant given that poor adherence (i.e., the degree to which a participant follows the recommendations of an intervention) and rates of attrition are known primary barriers to achieving optimal treatment outcomes for mental health worldwide[16,17]. International evidence on the effectiveness of interventions for suicidal people in custody (e.g., multicomponent programs, group-based programmes, and peer-support interventions) show many gaps in knowledge and limited use of high-quality research designs such as randomised controlled trials[18]. Reviews of risk factors relating to suicide and self-harm behaviour suggest that interventions should encourage social structures, develop positive relationships and provide purposeful activity by encouraging interventions that utilize a holistic approach to supporting the mental health of prisoners[3,19].

In the UK prison service and the Polish penitentiary service, cognitive behavioural programmes have been used for many years to address offending behaviour [20,21,22]. One element of such programmes includes the use of social problem-solving. In the community, meta-analytical reviews of problem-solving skills show beneficial impact of reducing symptoms of suicidal ideation (MD -1.58, CI 95% -1.58 to -0.44), outcomes of repetition of self-harm at 4 months (OR 0.65, CI 95% 0.36 to 1.16) and final follow-up (OR 0.76, CI 95% 0.55 to 1.05)[23]. Theorists[24] note that individuals with a characteristic set of negative thoughts and feelings about problems tend to blame themselves for problems and doubt their own ability to solve problems effectively. Avoidant and impulsive responses result in, in-effective problem-solving and thus risk reinforcing the negative beliefs and feelings that impact on mental health and well-being[25]. Use of problem-solving skills is important for many who display symptoms of depression, self-harm, or violent behaviour because they often cite a problem as the main cause[26].

In the UK prison service, a co-produced manualised problem-solving training package (consisting of a workbook, digital materials, and handbook) for prison staff and prisoners was developed[27]. Comprising of five 3-hour group training sessions and using a six-step cognitive model targeting the following skills (e.g.,

For peer review only - http://bmjopen.bmj.com/site/about/guidelines.xhtml

supporting alternative styles of thinking; self-control; use of social perspective taking and consideration of consequences arising from problem-solving deficits). Key findings from research evaluating the package demonstrated a promising reduction in repeat self-harm behaviour[23]. However, problem-solving skills in Poland are not used with those who present with self-harm behaviour or concerns of mental health and well-being.

The study therefore aimed to adapt the UK problem-solving model using the Ecological Validity Model process with four stages: (1) a demonstration of the training materials to prison staff and an evaluation of the feasibility (whether something should be done, should we proceed with it and if so how) and acceptability (how well an intervention has been received by the target population), (2) a wider audit of the skills to gather feedback from the Polish prison community, (3) adaptation of the current training materials using culturally specific examples, use of forward and back-translation, and (4) two rounds of consultations to identify further amendments to the materials.

METHODS

Overall study collaboration

The study was conducted using a collaboration between: The Academy of Justice, in Warsaw, the University of Lodz, two Polish prisons (ZK Raciborz and ZK Klodzko), and the University of York (UK). The Academy of Justice convened the group of prison staff; identified participants for the wider audit; and liaised with prison staff at each prison site. One member of the research team (MZ) held a joint position with the University of Lodz. The Forensic Mental Health Group and PI (AP) from the University of York provided the original materials and was the project lead.

The Ecological Validity Process

An adapted Ecological Validity Model (EVM) [28] included an examination of the use of language, metaphors, and content (i.e., culturally appropriate, and syntonic language), the changing of case study scenarios (relevance and acceptability) and sought to maintain the fundamental therapeutic components of the intervention to retain the intervention comprehensibility and

For peer review only - http://bmjopen.bmj.com/site/about/guidelines.xhtml

completeness (Figure 1).

[INSERT FIGURE 1 HERE]

Knowledge user surveys

Three separate surveys were devised for stages 1,2 and 4 of the process. Survey one addressed the acceptability of the training and was evaluated using a brief [11-item] self-report questionnaire in November 2019. The questionnaire was aimed at identifying whether the skills were acceptable (how well an intervention has been received by the target population) and feasible (whether something should be done, should we proceed with it and if so how) to Polish prison officers. The questionnaire elicited a series of yes/no responses and semi-structured qualitative comments. Survey two informed a wider audit on the use of problem-solving skills within people who were working in the Polish criminal justice system using a 6 item self-report questionnaire during January and February 2020. Survey three (in round one of the consultations) was completed using an 8-item questionnaire; and in round two of the consultation, it was further extended to a 12-item questionnaire and was completed between May 2020 and August 2021.

PROCEDURE

Stage one: Demonstration of the training package for Polish prison staff

The problem-solving demonstration sessions were delivered by KM and AP in The Academy of Justice during a three-day period in November 2019. Ten prison staff members attended the training by invitation by The Director of the Academy of Justice and represented different roles across the Polish prison service. Apart from a personal willingness to participate staff had to meet two further criteria: (i) to have a good knowledge of English, and (ii) to work in a position where the skills could be used in the future. The sessions included a demonstration of the problem-solving skills, sharing of the UK case study examples and a series of role play exercises to support adaptation of the materials (see supplementary materials). At the end

For peer review only - http://bmjopen.bmj.com/site/about/guidelines.xhtml

of the training session, staff were asked to provide feedback using survey one.

Stage two: Audit of the problem-solving skills with staff working in the wider Polish prison community

The purpose of the audit was to gain a wider perspective and greater understanding from a larger population of people who either worked within the prison service or who were undergoing training to become a prison officer. During January and February 2020 MZ (a CBT psychotherapist in Poland with knowledge of the scheme) was trained by the PI (AP) in use of the intervention. MZ individually self-selected and approached students and staff to take part in the study. MZ demonstrated the problem-support skills with use of a brief 90 session that included an animation and teaching of the skills. Staff and students were showed copies of the training workbooks before completion of survey two.

Stage three: Adaptation of the materials into Polish

Feedback from stages one and two informed the translation of the training documents. The translation from English into Polish was completed by MZ. Back translation was completed independently by a translation service in the UK. In addition, the translation was checked against the original text by the project PI (AP). Ratification of the text was completed with further discussion with colleagues in Poland to ensure the wording and language was comparable and culturally appropriate [29].

Stage four: Consultation process of the adapted materials

Feedback from staff already engaged in stages 1 and 2 were asked to take part in two rounds of consultation. In round two, recruitment of a 12 prison officers from two Polish prisons (who hadn't seen the materials before) were asked to consult and review the translated training package to understand more about how prisoner officers perceived the package without prior knowledge of its development. During the approach by MZ, 10 prison officers declined to take part due to the 'pressures of daily duties'. The findings from the consultation process were categorised into changes relating to the EVM model including language, metaphone and content, relevance and acceptability and intervention comprehensibility and completeness.

For peer review only - http://bmjopen.bmj.com/site/about/guidelines.xhtml

Data Analysis

All survey results were anonymized and data were entered into a series of different Excel spreadsheets. IBM SPSS Statistics (version 26) and Statistica StatSOFT version 13 were used to explore the quantitative responses generating mean scores and descriptive statistical information. The distribution of the data were examined to identify use of either parametric or nonparametric testing at ($p<0.05$). The qualitative comments were summarized at each stage in the study, inductive coding was used to synthesise the evidence and then generate themes [30]. The COREQ checklist (http://www.equator-network.org/reporting-guidelines/coreq/) were reported to ensure transparency of the methodology. Coding was completed by MZ and AP generating themes. Qualitative comments within each theme were additionally 'counted' to provide a numerical frequency which enabled a direct comparison of comments within each theme. The responses from stage 2 were divided into those reporting either by Prison Staff (PS) or students (S) in training.

Patient and public involvement

People in custody were involved throughout the adaptation process.

RESULTS

Stage one: Demonstration of the training package for Polish prison staff

Characteristics of the training sample

Prison staff (n=10) were aged between 29 and 39 years of age (mean age 34.7 years, SD = 3.25). All identified as white European and spoke Polish as their first language. Most of the group were female 7/10 (70%); one participant considered themselves disabled. Participants had worked in the prison service for an average of 103.4 months (SD = 39.8, min = 51 months, max = 178 months), and within the current prison for a mean number of 65.7 months (SD = 35.5, min = 6,

For peer review only - http://bmjopen.bmj.com/site/about/guidelines.xhtml

max = 111). Prison staff had different roles, including psychologists, penitentiary departmental managers (providing substantive supervision over psychologists and educators) and specialists from the Central Board of the Prison Service. All staff reported dealing with an incident of self-harm or attempted suicide within an average of 16 months prior (SD = 15.8, min = 1 month, max = 36 months). Of those reported incidents 1/10 (10%), reported self-poisoning, 3/10 (30%) reported general self-injury four reported incidence of attempted suicide 4/10 (40%). Incidence of violence was reported by 7/10 (70%) of staff reported an experience of being involved in a violent incident that could have been avoided 3/10 (30%). Most prison staff 7/10 (70%) encountered prisoners with mental health problems, just under a quarter experienced these on a daily or weekly basis 2/10 (20%).

Acceptability of the training package and use of the skills

All participants thought that the training was useful, and most learned new skills (Table 1). Some of the main themes included: an opportunity to learn new skills ('new knowledge' and a 'different approach is always useful'), providing useful communication skills ('learning how to speak to prisoners'), developing reflection of perspective ('to view it through the prisoner's perspective'), use of a collaborative approach ('working towards a joint search for a solution, not imposing one views'), and use of open-ended questions to progress behaviour change ('work on changes in thinking is important'). Most staff felt that training could be delivered by prisoners, prison staff, psychologists, educators, and practitioners within the prison site. Half the group wanted the training to be longer and nearly all thought that an additional booster session would be useful.

Table 1: Results following demonstration of the training package for Polish prison staff

Questionnaire	n/10<br>(% yes)	Qualitative comments
In what ways was the training helpful to you?	NA	Development of a new perspective<br>A new technique/tool kit/ alternative approach

For peer review only - http://bmjopen.bmj.com/site/about/guidelines.xhtml

		Exchange of ideas between the UK & Poland
What are the positive aspects of the scheme?	NA	Straight forward, simple, easy to understand<br>Problems solved by prisoners<br>The development of coping skills<br>Group work<br>Providing a structure to help change
Is the training applicable for your job role?	10/10 (100%)	NA
Did you learn any new skills?	7/10 (70%)	NA
If yes, what new skills did you learn?	NA	Working from the prisoners perspective<br>How to speak to prisoners<br>Development of problem-solving skills
Who do you think is best to offer the training to prison staff or prisoners?	NA	Prisoners, prison staff, psychologists, educators, practitioners within the prison site.
Is the training relevant to your job role in the prison?	9/10 (90%)	NA
What improvements could be made to the scheme?	NA	Development of more practice exercises<br>Use of additional case study examples<br>Development of the materials
Is the length of the training adequate?	5/10 (50%)	The training should be longer.
Would it be useful to have a booster session?	9/10 (90%)	NA
How will the training be sustained?	NA	In everyday use<br>Training of prisoners on an individual basis<br>Training as part of prisoner officer rules

Stage two: Audit of the problem-solving skills with staff working in the wider Polish prison community

Characteristics of the audit sample

For peer review only - http://bmjopen.bmj.com/site/about/guidelines.xhtml

A volunteer sample of 67 persons took part in the wider audit: 39/67 (58%) were prison staff and 28/67 (42%) were students. Of the prison staff 30/39 (77%) identified as male. The age ranged from 20 to 50 years of age; 12/39 (31%) worked as Educators. Other staff (3/39, 7%) worked in Prison Management, 15/39 (38%) in the Security Department, 10/39 (25%) as a Shift Commander; 1/39 (2%) in the Finance Department, 2/39 (5%) in the Quartermaster Department, 3/39 (7.8%) in the Therapeutic Department; and 2/39 (5%) persons defined themselves as an Inspector. Students were aged between 20 and 30 years of age: 9/28 (32%) were Male. All students were working towards a Master's degree in National Security with an aim to enable them to join the prison service.

Overall results from the audit

Many prison staff and students thought that problem-solving skills should be taught as part of standard training and thought there were benefits to using the skills 53/67 (80%) (Table 2). The perceived benefits were wide ranging. Prison staff were more likely to recognise use of the skills as an opportunity to change behaviour, to empower others to increase the pace of decision making 28/67 (42%), develop knowledge 30/67 (45%) and apply the skills in instances of crisis management 18/67 (27%).

Students were most likely to report use of the skills in general problem solving 22/67 (33%). Staff were more likely that students (37% vs 11%) to use the skills for self-development and knowledge. Students were more likely than staff (35% vs 15%) to use the skills to develop their own coping strategies. Over half of prison staff 23/39 (58%) and most students 19/28 (70%) saw relevance of the skills within their job role. No significant difference were found in the opinion of students or staff in the perceived beneficial of integrating the skills into the training scheme for newly qualified prison officers ($X^2(1, N = 66) = 3.45, p > 05.$). Prison officers when compared to students were significantly more likely to accept that the training could be translated into skills for prisoners (U=51, median rank: 243; 222), p<0.05.

Table 2: Audit of the acceptability of the problem-solving skills with a wider Polish prison community

For peer review only - http://bmjopen.bmj.com/site/about/guidelines.xhtml

Questionnaire items	PO (N% yes)	Student (N% yes)	Prison Officer (PO) and Student (S) Qualitative themes	
Do you think problem-solving skills should be taught to prison officers as part of standard training?	38/39 (97%)	23/27 (85%)	NA	NA
Do you think there are benefits from learning the skills?	34/39 (87%)	24/27 (88%)	NA	NA
If yes, what are the benefits?	NA	NA	Changing behaviour (PO=9/33 27% vs S=2/24 8%)<br>Crisis planning & prevention (PO=2/33 6% vs S=3/24 12%)<br>Developing coping strategies (PO=2/33 6% vs S=3/24 12%)<br>Developing personal skills (PO=1/33 3% vs S=1/24 4%)<br>Developing social networks (PO=4/33 12% vs S=1/24 4%)<br>Generating solutions (PO=1/33 3% vs S 1/24 4%)<br>Helping someone (PO=0/33 0% vs 5/24 20%)<br>General problem solving (PO=2/33 6% vs S=8/24 33%)<br>Empowerment to make decisions at pace (PO=14/33 42% vs 2/24 8%).	
What do you like most about the idea of using the skills?	NA	NA	Ability to compromise (PO=3/32 9% vs S=0/17 0%)<br>Knowledge & self-development (PO=12/32 37% vs S=2/17 11%)<br>Developing coping strategies (PO=5/32 15% vs S=6/17 35%)<br>Empowerment (PO=6/32 18% vs S=5/17 29%)<br>Tailored to individual need (PO=1/32 3% vs S=0/17 0%)<br>Improved communication (PO=1/32 3% vs S=2/17 11%)<br>Accessible to help others (PO=4/32 12% vs S=2/17 11%)	
Could you see them being used in your job role?	23/39 (58%)	19/27 (70%)	NA	NA

For peer review only

For peer review only - http://bmjopen.bmj.com/site/about/guidelines.xhtml

If yes, in what capacity?	NA	NA	Solving problems (PO=1/22 4% vs S=3/16 18%)<br>Helping others (PO=0/22 0% vs S=3/16 18%)<br>Applicability in life experiences (PO=0/22 0% vs S=2/16 12%)<br>Developing empathy (PO=0/22 0% vs S=2/16 12%)<br>Crisis management (PO=6/22 27% vs S=4/16 25%)<br>Increased knowledge (PO=10/22 45% vs S=1/16 6%)<br>Empowerment (PO=3/22 13% vs S=1/16 6%)<br>Social cultural activities (PO=2/22 4% vs S=0/16 0%)

Stage three: Adaptation of the materials into Polish

Feedback from stages 1 and 2 allowed the materials to be translated into Polish using a series of changes within the structure of the EVM. These included:

Language, metaphors, content

Words were added to the workbooks (e.g., 'to be' or 'to have') to make the context more understandable in Polish. Words were also changed to reflect different cultural differences. In addition, some English words were non-existent in Polish and in these cases similar words were found and replaced to ensure that the comprehension of the materials remained the same.

Relevance and acceptability

One male case study was reviewed as part of the training package (supplementary materials). Changes to the case study ensured its applicability to the Polish penitentiary system. Changes to the context and background information of the case study reflected the daily routine of prisoners and signposted resources in Poland. Options generated from the case study that did not exist within the Polish context were changed to reflect forms of assistance available in Poland.

Intervention comprehensibility and completeness

The workbook titles were changed to reflect the comments made during stages 1 and 2. The

For peer review only - http://bmjopen.bmj.com/site/about/guidelines.xhtml

theoretical underpinning of the intervention was adhered to throughout the structure of the workbooks to ensure that the intervention comprehensibility was maintained. British names of resources were changed to Polish resources to enable the completeness of the intervention to be supported.

Stage four: Consultation process of the adapted materials

Characteristics of the sample: first and second consultations

Consultation of the adapted materials included two rounds. Round one included (i) prison staff from stage one 8/10 (80%), and a (ii) wider group of professionals 10/39 (25%) from stage two. Round two included those participants who took part in round one plus a group of serving prisoner officers (n=12) who had not previously seen the materials (Table 3).

In round one the results showed that most people (83%) liked or really like the pictures and diagrams, 89% reported both that the language reflected the context of the prison environment; and the skills were perceived as useful within daily work. 100% of participants reported that they would like to attend training.

Table 3: Consultation process of adapted materials round two

Second consultation (n=28)	Definitely not (yes)	Rather not (yes)	No strong preference	Rather (yes)	Definitely (yes)
Quality	0	1 (4%)	2 (7%)	15 (53%)	10 (36%)
Clarity	0	0	5 (18%)	12 (43%)	11 (39%)
Diagrams	0	1 (4%)	2 (7%)	13 (46%)	13 (46%)
Structure	0	2 (7%)	2 (7%)	15 53%)	8 (29%)
Materials	0	0	4 (14%)	17 (60%)	6 (21%)
Training modules	0	2 (7%)	2 (7%)	14 (50%)	9 (32%)
Understanding clarity and content	0	1(4%)	5 (18%)	14 (50%)	7 (25%)

For peer review only - http://bmjopen.bmj.com/site/about/guidelines.xhtml

Acceptability of the case study	1 (4%)	1 (4%)	2 (7%)	15 53%)	8 (29%)
Language adaptation	1 (4%)	1 (4%)	2 (7%)	14 (50%)	10 (36%)
Possibility of self-development	1 (4%)	1 (4%)	5 (18%)	18 (64%)	3 (11%)
Development of coping strategies	0	2 (7%)	3 (11%)	14 (50%)	8 (29%)
Level of innovation: use within the system	0	1 (4%)	3 (11%)	14 (50%)	9 (32%)

Language, metaphors, content

The combined results from the qualitative consultations in rounds one and two showed that overall, the language used were appropriate for the prison context ('I rate the materials at a level appropriate to the subjects' language. 'I think it is understandable and also simple'). The second consultation process did identify some useful further iteration on use of appropriate language to ('unify terminology' and reflected that 'some words are not good in non-changed from original version form'). Based on this feedback a final edit of the materials were completed.

Relevance and acceptability

The workbooks contained diagrams and pictorial representations through cartoon like characters. These were used in the original UK workbooks to aid literacy levels[31,32]. In Poland, most prison staff thought these were acceptable with wider benefit to other client groups recognized ('the presented training materials are interesting their great value is universalism'; they can be implemented with different groups of people'). The general opinions about the overall presentation of the workbooks were good ('diagram and pictures facilitate understanding of the presented content').

DISCUSSION

To our knowledge this study is the first to demonstrate use of the EVM to develop the cultural adaptation of an evidenced-based UK problem solving skills training package for prison staff and

For peer review only - http://bmjopen.bmj.com/site/about/guidelines.xhtml

prisoners at risk of self-harm, mental health, and well-being for use in Poland. Cultural adaptation procedures are key in the acceptability and recognized feasibility of using any intervention[12]. Iterative input from Polish prison staff was vital in generating a training package with the potential to support good adherence.

The results demonstrated acceptance of the skills regardless of staff or student group, indicating that the use of the skills had potential widespread appeal across the Polish penitentiary system. Similar findings to use of the package in the UK were reported by prison staff and prisoners[24]. As expected, staff in comparison to students could envisage greater application of the skills within the prison context. This may well reflect their experience of working within the prison environment. The guiding principle of cultural adaptation is to work closely with stakeholders during early developmental stages to ensure that they can be sustained [32-33]. Prisons are challenging settings, trust, confidentiality, and fairness are more difficult to establish in prison settings[34]. Failure to acknowledge something as acceptable or feasible is a barrier to implementation and wide-spread use. Interventions that engage staff from across the prison sites help consider the integration and development of sustainable models to prevent self-harm behaviour and promote mental health and well-being going forward.

Limitations

The study has several limitations. First, the volunteer self-selecting nature of the sampling is unlikely to reflect the representative nature of all staff in the Polish prison service. Arguably, the nature of the sampling within this evaluation may impact on the generalisability of the training package. Extensions of this work would require feedback from a diverse range of prisoners and use of novel participatory strategies. Although feedback from staff reported that the skills would be relevant in circumstances of 'crisis management'; further evaluation of the impact of the training package should also gather views on the perceived impact on the management of suicidal and self-harm behaviour.

Implications and future research

For peer review only - http://bmjopen.bmj.com/site/about/guidelines.xhtml

Our adaptation processes resulted in good levels of acceptability and feasibility, with positive feedback from prison staff and students. Recommendations refer to the necessity of using problem-solving skills as a part of standard training, by integrating skills such as role-play to support communication skills. Further development of the workbook and training package need to consider adaptations to ensure that the materials are widely accessible to prisoners with different protected characteristics. The inclusion of further narrative case studies for females in prison would require additional consideration.

CONCLUSION

The process of cultural adaptation enables researchers to efficiently tailor evidence-informed interventions for diverse populations while retaining the core components that underpin efficacy. The EVM provides a useful framework for adapting interventions. Use of this method produced an acceptable training package for the Polish prison service. Further evaluation of the effectiveness of the problem-solving training skills needs to be explored using a randomised controlled trial design.

DATA AVAILABILITY STATEMENT

Data is made available upon reasonable request.

ETHICS STATEMENTS

Ethical approval for the study was granted by the Department of Health Sciences at the University of York in the UK and reviewed by a RODO Data Protection Specialist in The Academy of Justice, in Warsaw, Poland. Permission was granted from each prison in taking part in the study. All study participants received full informed consent.

ACKNOWLEDGEMENTS

With many thanks to the UK HMPPS Intervention services, to all the prison staff that contributed to the project including the prisons of ZK Racibórz and ZK Kłodzko. To the Central Board of Prisons in Poland who gave permission for this research.

For peer review only - http://bmjopen.bmj.com/site/about/guidelines.xhtml

FUNDING

This study was funded through the Wellcome Trust, Centre for Future Health, Rapid Response Fund, University of York, UK.

AUTHOR CONTRIBUTION STATEMENT

AP and MZ devised the study with the initial support from PL. AP and KM delivered the training in stage one. JR conducted the analysis and BNM supported Polish colleagues in the written work for the publication. All elements of the project were overseen by AP. All authors worked on the draft of the manuscript.

REFERENCES

1. Durcan, G. (2021). The future of prison mental health care in England. M Centre for mental health. [CentreforMentalHealth_TheFutureofPrisonMentalHealthCare_0.pdf]. Accessed September 2022.

2. Fazel, S., Hayes, A. J., Bartellas, K., Clerici, M., & Trestman, R. (2016). Mental health of prisoners: prevalence, adverse outcomes, and interventions. Lancet Psychiatry, 3, 871-881 https//doi: 810.1016/S2215-0366(1016)30142-30140. Epub 32016 Jul 30114. PMID: 27426440; PMCID: PMC25008459.

3. Favril, L., Yu, R., Hawton, K., & Fazel, S. (2020). Risk factors for self-harm in prison: a systematic review and meta-analysis of 35 studies covering 663735 adults. Lancet Psychiatry, 7(8), 682-691. https//doi: 610.1016/S2215-0366(1020)30190-30195.

4. Brugha, T., Singleton N, Meltzer H, Bebbington P, Farrell M, & Jenkins R, G., L. (2005). Psychosis in the community and in prisons: a report from the British National Survey of psychiatric morbidity. American Journal of Psychiatry, Apr;162(4):774-80. https//doi: 10.1176/appi.ajp.162.4.774. PMID: 15800152. 774-780.

5. Favril, L., Indig, D., Gear, C. *et al.* (2020) Mental disorders and risk of suicide attempt in prisoners. *Soc Psychiatry Psychiatr Epidemiol* **55**, 1145–1155. https://doi.org/10.1007/s00127-020-01851.

6. Favril, L., Shaw, J., Fazel, S. (2022). Prevalence and risk factors for suicide attempts in prison, *Clinical Psychology Review, Volume 97, https://doi.org/10.1016/j.cpr.2022.102190.*

For peer review only - http://bmjopen.bmj.com/site/about/guidelines.xhtml

7 Favril, L., O'Connor, R., Hawton, K., & Vander Laenen, F. (2020). Factors associated with the transition from suicidal ideation to suicide attempt in prison. *European Psychiatry, 63*(1), E101. https//doi:10.1192/j.eurpsy.2020.101.

8. Vos, T., Lim, S. S., Abbafati, C., Abbasi, M., & Abbasi-Kangevan, M. e. a. (2020). Global burden of 369 diseases and injuries in 204 countries and territories, 1990-2019: a systematic review analysis of the Global Burden of Disease Study 2019. The Lancet, 396(10258: 1204-1222: doi), 1204-1222: https//doi.org/1210.1016/SO1140-6736 (1220) 30925-30929.

9. Ministry of Justice 2022 Safety in Custody Statistics, England and Wales: Deaths in Prison Custody to December 2017 Retrieved from National Statistics: Ministry of Justice, 2022: https://www.gov.uk/ /collections/safety-in-custody-statistics. Accessed April 2023.

10. Lizińczyk, S. (2023). Characteristics of suicides committed in Polish prisons, 2015–2019: *Archives of Criminology*, (XLII/2), 313–336. https://doi.org/10.7420/AK2020P

11. Przybyliński S., Marczak M. (2008). Self-mutilation among female and male prisoners. In: W. Ambrozik, H. Machel, P. Stępniak (eds.) The mission of the Prison Service and its tasks in relation to the current penal policy and social expectations. IV Polish Penitentiary Congress (p. 455) Poznań-Gdańsk-Warsaw: UAM, UG, Pedagogium WSPR and CZSW.

12. Culbert, G.J. & Williams, A. B. (2018). Cultural Adaptation of a Medication Adherence Intervention With Prisoners Living With HIV in Indonesia: A Pragmatic Approach to Intervention Development. Journal of the Association of Nurses in AIDS Care, 29, Issue 3, Pages 454-465,ISSN 1055-3290, https://doi.org/10.1016/j.jana.2017.11.006.

13. Thakker, J. (2014). Cultural factors in offender treatment: Current approaches in New Zealand. Social and Behavioural Sciences, 113, 213-223. https//DO 10.1016/j.sbspro.2014.01.028.

14. Castro, F. G., Barrera, M. J., & Holleran Steiker, L. K. (2010). Issues and challenges in the design of culturally adapted evidence-based interventions. Annual Rev Clin Psychology 6:213-39. https//doi: 10.1146/annurev-clinpsy-033109-132032.

15. Faregh, N., Lencucha, R., Ventevogel, P., Dubale, B. W., & Kirmayer, L. J. (2019). Considering culture, context and community in mhGAP implementation and training:

For peer review only - http://bmjopen.bmj.com/site/about/guidelines.xhtml

challenges and recommendations from the field. Int J Ment Health Syst 13:58. https//doi: 10.1186/s13033-019-0312-9.

16. Reingle Gonzalez, J. M., & Connell, N. M. (2014). Mental health of prisoners: identifying barriers to mental health treatment and medication continuity. American journal of public health, 104(12), 2328-2333. https://doi.org/10.2105/AJPH.2014.302043.

17. Nagpal, T.S., Mottola, M.F., Barakat, R., Prapavessis, H. (2021). Adherence is a key factor for interpreting the results of exercise interventions*, Physiotherapy, Volume 113, Pages 8-11, https://doi.org/10.1016/j.physio.2021.05.010.*

18. Carter, A. Butler, A. Willoughby, M. Janca, E., Kinner, S.A., Southalan, L., Fazel, S. Borschman, R. Interventions to reduce suicidal thoughts and behaviours among people in contact with the criminal justice system: A global systematic review. *E ClinicalMedicine 2022;44: 101266 Published online 14 January 2022 https://doi.org/10.1016/j.eclinm.2021.101266.*

19. Perry, A. E., (2020). Self-harm in prisons: what do we know and how can we move forwards? www.thelancet.com/psychiatry Vol 7 August 2020, 649-650. https://doi.org/10.1016/S2215-0366(20)30298-4.

20. Gobbett MJ, & Sellen, J.L. (2014). An evaluation of the HM prison service thinking skills programme using psychometric assessments. Int J Offender Therapy Comp Criminol., Apr;58(4):454-73. https//doi: 10.1177/0306624X12472485. Epub 2013 Feb 6. PMID: 23390065.

21. Majcherczyk, A. (2011). Selected rehabilitation programs - theoretical and practical perspective. *Przegląd Więziennictwa Polskiego", nr 70, p. 5-30.*

22. Rejzner, A., & Szczepaniak, P. (2009). Therapy in rehabilitation, volume I Theoretical approach & volume II A practical approach. Warszawa: Żak.

23. Perry, A. E., Waterman, M., Dale, V., Moore, K., & House, A. (2021). The effect of a problem support intervention on self-harm and violence in prison: an interrupted time series analysis using routinely collected prison data. EClinical Medicine- The Lancet, 32 (1007021), 1-11 https://doi.org/10.1016/j.eclinm.2020.100702.

For peer review only - http://bmjopen.bmj.com/site/about/guidelines.xhtml

24. D'Zurilla, T. J., Chang, E. C., Nottingham, E. J. IV, & Faccini, L. (1998). Social problem-solving deficits and hopelessness, depression, and suicidal risk in college students and psychiatric inpatients. *Journal of Clinical Psychology, 54*(8), 1091–1107.https://doi.org/10.1002/(SICI)1097-4679(199812)54:8<1091::AID-JCLP9>3.0.CO;2-J.

25. D'Zurilla, T. J., & Goldfried, M. R. (1971). Problem solving and behavior modification. *Journal of Abnormal Psychology, 78*(1), 107–126.https://doi.org/10.1037/h0031360.

26. Linehan, M. M., Camper, P., Chiles, J. A., Strosahl, K., & Shearin, E. (1987). Interpersonal Problem-Solving and Parasuicide. Cognitive Therapy and Research, 11(1), 1-12. https//Doi 10.1007/Bf01183128.

27. Perry A. E., Waterman MG, House A, *et al* Problem-solving training: assessing the feasibility and acceptability of delivering and evaluating a problem-solving training model for front-line prison staff and prisoners who self-harm *BMJ Open* 2019;**9:**e026095.https//doi: 10.1136/bmjopen-2018-026095.

28. Bernal, G., Bonilla, J., & Bellido, C. (1995). Ecological validity and cultural sensitivity for outcome research: issues for the cultural adaptation and development of psychosocial treatments with Hispanics. J Abnorm Child Psychol 23(1):67-82. https//doi: 10.1007/BF01447045.

29. Ozolins, U., Hale, S., Cheng, S. X., Hyatt, A., & Schofield, P. T. (2020). Translation and back-translation methodology in health research - a critique. Expert Review of Pharmacoeconomics & Outcomes Research, https//DO 10.1080/14737167.2020.1734453.

30. Braun, V., & Clarke, V. (2013). Successful qualitative research: A practical guide for beginners. London: Sage.

31. Morken, F., Jones, L. Ø., & Helland, W. A. (2021). Disorders of Language and Literacy in the Prison Population:A Scoping Review. Educ. Sci., 11, 77. https://doi.org/10.3390/ educsci11020077. Najwyższa, I. K. (2020). Security of inmates. Information on the inspection results. KPB.430.002.2020, Nr ewid. 52/2020/P/19/040/KPB, available online: https://www.nik.gov.pl/plik/id,22869,vp,25572.pdf [Accessed January 2022].

32. Castro, F. G., & Yasui, M. (2017). Advances in EBI development for diverse populations: towards a science of intervention adaptation. Prevention Science, 1-7. https// doi:

For peer review only - http://bmjopen.bmj.com/site/about/guidelines.xhtml

10.1007/s11121-017-0809-x.

33. Williams, A. B., Wang, H., Burgess, J., & X., L. (2013). Cultural adaptation of an evidence-based nursing intervention to improve medication adherence among people living with HIV/AIDS (PLWHA) in China. International Journal of Nursing Studies, 50(4):487-494. https// doi: 10.1016/j.ijnurstu.2012.08.018.

34. Azbel, L., Grishaev, Y., Wickersham, J. A., Chernova, O., Dvoryak, S., Polonsky, M., & Altice, F. L. (2016). Trials and tribulations of conducting bio-behavioral surveys in prisons: implementation science and lessons from Ukraine. International Journal of Prisoner Health, 12(2):78-87. https//doi: 10.1108/IJPH-10-2014-0041. 25245352.

For peer review only

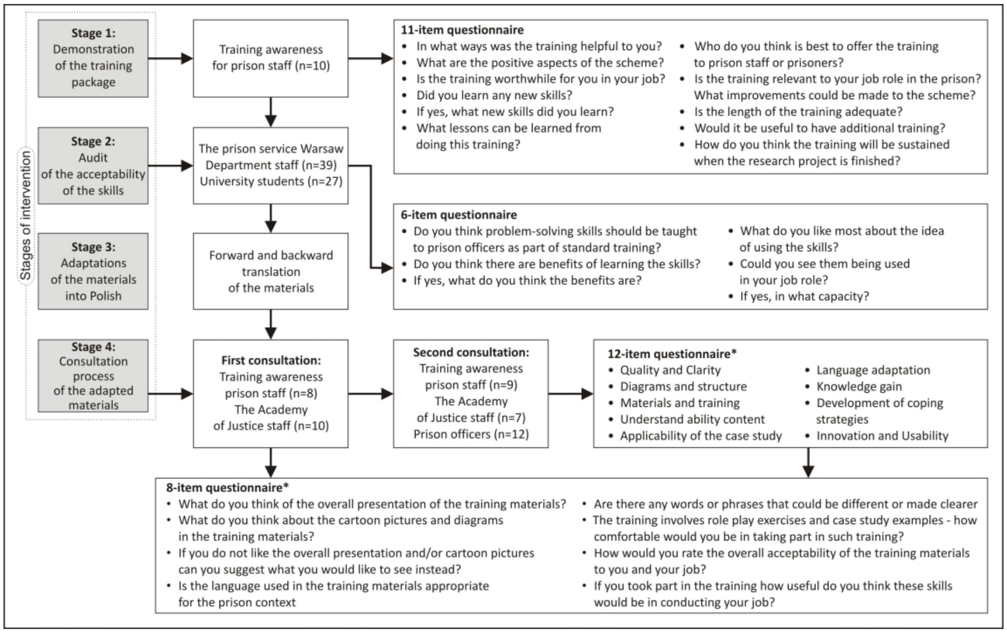

Figure 1: Study Flow Process

263x164mm (300 x 300 DPI)

Supplementary File

Session one: Role play example – demonstrating how a negative attitude can affect the outcome of a problem

- Facilitator introduce the first role-play example to show about people behave and react to a situation can vary depending upon how they feel:

'That you are on your way to an interview in your car and you suddenly realised that you have t wo but two flat tyres. You suspect that a neighbour has let your tyres down. All dressed up in a suit and tie. You have to stop I the middle of the road blocking the traffic which grinds to a halt behind you which starts causing chaos.' A bus stop is near by. Someone in another car stops and laughs at you – pulls over the stops you go over to them…

- Facilitators to demonstrate the scenario

- Facilitator to question the group:
  - How did the driver feel about the situation?
  - What was there attitude like?
  - Did the incident end in a positive way?
  - What could they have done differently/said?

Facilitator to encourage the group to mimic the role-play exercise so…

- Ok so I want you to get into pairs. One person be the driver who approaches the person who has laughed at them. I want you to start your conversation telling the person how you feel what your problem is and see how they respond.
- When we have done this we are going to go around the room and talk about what happened in each person's situation – was the problem resolved- did the person help – did they get to the interview or not?
- Then repeat the exercise and role play it with a positive attitude. The exercise shows that if you do things in the right way you can often get a more satisfactory outcome.

On a flip chart and with the whole group the facilitator uses the example to demonstrate the problem-solving steps.

For peer review only

Steps in the model:

Step1: Getting the right attitude

Step 2:Reflect and recognise triggers – facilitator notes that if people reflect on previous incidents that didn't turn out as anticipated this can help to identify how they felt at the time, what they thought and how they behaved. Recognising these triggers can play an important role in behaviour change.

Step 3: Define the problem clearly – often we don't have all the information we need to resolve a situation and sometimes you might use these skills to help support how someone feels to enable them to cope better with a situation. Facilitator to prompt – what information did the person does not have in the car scenario. Using what/where/when/why type questions can help to elicit this information in more detail. This specific information helps someone to explore the crux of the problem. To use the problem-solving skills effectively a problem needs to be clearly defined. For example, 'I have a problem with my wife' isn't a problem that is sufficiently defined to be able to use the skills.

Step 4 Brainstorming solutions – Once we have as much information as possible and a clear problem definition, we encourage people to think of as many different solutions (good or bad) to help promote ideas that the person might want to try out. In this model of problem solving, it is important that the person comes up with their own ideas (see the list of suggestions in Appendix 1) if someone requires some support to do this.

Step 5 Decision making – For each solution we then make a list with the possible pros and cons of each idea. It is good to go through the 'not so positive ideas' as it allows someone to explore what might go wrong is they choose an option that is less than favourable. This process helps someone to shortlist the ideas to two or three that they wish to pursue and take forwards into an action plan.

Step 6 Make a plan -Then we think about making a plan – this can be a different plan for everybody [the right plan for one person might not be the case for someone else]. Write down your plan – what would you do? What is your plan? The more specific the plan is the more likely it will work. In what order are you going to do the tasks? When are you going to do each task? Do you need some help or support to complete the task?

Step 7 Review – Problem-solving is an iterative process and not always does it work for many logistical reasons. It is therefore sensible to support an individual to review what happened. Did your plan work? What went wrong? Using the skills again to produce another alternative and try again using the skills. The more someone practicing the skills the better they get at using them.

For peer review only

Male Case Study Example:

James is 22 years old. He is one of six siblings and lives with his father and stepmother. James's relationship with his parents was troubled from a young age and his father would come home drunk and beat James. His stepmother found it difficult to deal with James's aggressive emotional outbursts and he was excluded from school at age 11 for poor behaviour and angry outbursts.

James started to mix with a gang of older boys who were known in the area for committing petty crimes. He became involved in drugs at age 13 years and was caught by the police for burglary when he was 16 years old. He also had a series of relationships with older women which led to a number of pregnancies resulting in two sons and a new baby. James's stepmother was unable to control his behaviour and did not want him in the house anymore so James was asked to leave.

James went to stay with a friend but soon ended up living in a hostel. He found it difficult to get a job and ended up stealing to support his drug habit. His physical and mental health deteriorated and he no longer took care of himself. One day he took drugs and alcohol and ended up in the Accident and Emergency department at the hospital.

James was finally convicted for a series of burglaries and ended up in prison for the first time. At an all-time low James has contact with his family, with his partner visiting him with the kids on a regular basis. James regularly self-harmed when he was feeling particularly stressed in prison. James was placed in a shared cell and though having settled into prison life James felt angry and frustrated.

On a recent prison visit his partner told him that the council were planning to change their accommodation because James was no longer living with them. The change in circumstances would mean that James's partner could be moved outside of the local area. James returned to the wing in a low mood. He feels inadequate and powerless to do anything about the change in circumstances. James 'kicks off' in his cell.

Recognising and Identifying Problems

Facilitators encourage the group to use the 7-step model to work through the case study example.

For peer review only

Re-visit case study

James felt daunted by trying out brainstorming. At first he felt that nothing would help the situation. As James and his prison officer started to work together he gained momentum with the situation and provided a number of different ideas which helped him feel more in control of the situation.

'my partner is being forced to move out of her house and is being moved away from the area and I will not see my family every week at visiting time'

Here are some practical solutions

Ask my partner to ring the council and find out where she is moving to

Arrange a specific time when they will be able to visit so I can look forward to the visit

Get some photos of my baby and partner to put up in my cell

Ring my partner more often

Talk to visits to see if I can get extended visiting time when they come

Here are some emotional solutions

Focus on keeping myself to myself and not getting into trouble whilst in prison

Plan the time I have in prison to keep me busy

Ask if I can have extra jobs to do in the prison to keep my mind occupied

Go to the prison gym and take part in some exercise

Consider going to education to see if they have any courses I can attend

For peer review only

Re-visit the case study

When James looked at the initial selection to her brainstorm he decided to cross out. He thought they lacked feasibility and had a negative effect on his wellbeing.

The James grouped the remaining ideas. She grouped them into three categories:

Activities to keep himself busy

Methods of organising time

Strategies to manage stress

After thinking about the advantages and disadvantages of these possibilities James decided that planning time in advance had the advantage of reducing his stress. James solution was first to speak to his personnel officer about contacting education and the jobs section within the prison to find out more about what opportunities were available to him.

James also wanted to ring his partner to discuss the move location and ask her to send some pictures of the baby for his cell wall.

Overall, James thought the combination of these two strategies had a good chance of allowing him to feel better about his time in prison and seeing his family less.

SMART action plan and reviewing progress

SMART – Action plan

**Specific**	clear definition of what the action is
**Measurable**	it can be clearly determined whether or not the action has been achieved
**Achievable**	it is important to break the steps down so they can be successfully completed
**Relevant**	action is linked to the problem
**Time-bound**	timescale by which the action will be achieved

Re-visit the case study

James had to create two action plans one for each solution. They contributed to solving the same problem and could be implemented at the same time.

Extra activities in prison action plan

James decided he would ask his personnel officer about how to approach education and the jobs department in the prison. Once he had done this and gathered some more information James discovered that he needed to complete a jobs request card and place it in the post-box on the wing. He also discovered that he could go to education and speak with one of the teachers directly about what was available. In the next few days James identified the next tasks:

1. He considered his job options and also the potential demand to do such jobs within the prison. He wanted to choose some jobs that he would be allocated quickly, maybe because other people did not want to do them.
2. He decided to speak to his personnel officer again and other prison inmates to try and assess which job options he might have most success with.
3. Once he had gained the information he made a decision to put down a range of job choices with the hope of him being allocated something. These included working in the kitchen and canteen, cleaning on the wings and the toilet areas, working in the workshop making prisoner uniform for other prisons.
4. He was told that he would hear back about his choice of jobs within one week
5. He also planned a visit to education to discuss his course options

For peer review only

Further practice role play examples:

David has been on the wing for 12 months and has become addicted to drugs referred to as SPICE – on the last occasion he only narrowly escaped death and the healthcare staff had to bring him round. They showed him a video of himself and what had happened. He was completely shocked he had no idea how it had affected him and those around him. The video showed him on the floor with the paddles attached. Some members of staff on the video were visibly upset. David decides that this must stop but he doesn't know where to start.

Shane is 17 years old he has been convicted of robbery when he got involved with a gang of youths. He used to live with his grandma and dad who come to visit him regularly. One week his dad comes along to visit him without his grandma to tell him that his grandma has suddenly died from a heart attack. Shane was devastated he did not know how to handle the news – he goes back to his cell and wrecks his pad – and he is taken to the segregation unit to calm down. When he returns to the wing Shane is determined to get himself back on track, he is worried about whether he will be able to attend the funeral and what he can do to support his Dad.

Joseph an older man worked in banking industry for 25 years. He was finally convicted for fraud after a lengthy case and Joseph was sent to prison for 9 years. He was shocked by his incarceration and having been in Leeds for a few months he has been transferred to another prison, but he has struggled to adjust to prison life. He feels completely out of place. He looks like he is not looking after himself – his son comes to visit – he is ashamed of his father's behaviour and tells him he does not want to be part of his life anymore. A member of staff identifies Joseph as needing some help and refers him to you to see how best Joseph can be supported. Joseph feels isolated, alone, and angry, he refuses to come out of his cell when asked to by a member of staff.

For peer review only

Before Patrick came into prison my car was stolen. Patrick was recalled into prison on licence for not following the rules. On the night before he was arrested, he was involved in a car incident which meant that my car was stolen. He doesn't know what has happened to it. He is still paying road tax – He doesn't know if it is still OK or what to do about the insurance. He needs his car on the outside because he is a taxis driver – He is worried that when he is released, he won't be able to work and he won't have any money. He is talking to other prisoners to see if 'one of the boys' on the outside might be able to help, but the problem seems too big he wants to ignore it and pretend it hasn't happened. He thinks he can do nothing about his problem whilst in prison.

Andrew has a long history of self-harm behaviour and he has been monitored by staff for his self-harm behaviour for many months. Andrew has self-harmed himself ever since being in prison. Andrew is quite quiet and generally keeps himself to himself – he has a family and two children outside and he hasn't been able to pay the mortgage on the house for two months. The families' savings have all run out. Last week his wife came to visit in tears saying that she was worried that the house would be re-possessed. This is Andrew's main concern now. He feels completely out of control, he is panicking about what he can do to help support his family and his wife. He doesn't have a clear plan in is head about how he can help to resolve the situation and is feeling very anxious he feels like he needs to advise his wife on what they should do but thinks that time is running out for them. He blames himself for the situation and has been more reluctant to come out of his cell and go to work.

For peer review only

Consolidated criteria for reporting qualitative studies (COREQ): 32-item checklist

No	Item	Guide questions/description	Page numbers where marked in manuscript
**Domain 1: Research team and reflexivity**			
Personal Characteristics			
1.	Interviewer/facilitator	Which author/s conducted the interview or focus group?	Author MZ
2.	Credentials	What were the researcher's credentials? *E.g. PhD, MD*	Page 7
3.	Occupation	What was their occupation at the time of the study?	Page 7
4.	Gender	Was the researcher male or female?	Inferred by use of initials; MZ on page 7
5.	Experience and training	What experience or training did the researcher have?	Page 7 inferred by reference to qualification
Relationship with Participants			
6.	Relationship established	Was a relationship established prior to study commencement?	Page 6 under sub-heading 'study collaboration'
7.	Participant knowledge of the interviewer	What did the participants know about the researcher? e.*g. personal goals, reasons for doing the research*	Not applicable
8.	Interviewer characteristics	What characteristics were reported about the interviewer/facilitator? e.g. *Bias, assumptions, reasons and interests in the research topic*	None reported
**Domain 2: study design**			

No	Item	Guide questions/description	Page numbers where marked in manuscript
Theoretical framework			
9.	Methodological orientation and Theory	What methodological orientation was stated to underpin the study? *e.g. grounded theory, discourse analysis, ethnography, phenomenology, content analysis*	Page 8 and see Reference 30.
Participant selection			
10.	Sampling	How were participants selected? *e.g. purposive, convenience, consecutive, snowball*	Pages 7-8
11.	Method of approach	How were participants approached? e.*g. face-to-face, telephone, mail, email*	Pages 7-8
12.	Sample size	How many participants were in the study?	Pages 9-10 and in the Abstract
13.	Non-participation	How many people refused to participate or dropped out? Reasons?	Page 8 and under sub-heading 'stage four….'
Setting			
14.	Setting of data collection	Where was the data collected? e.*g. home, clinic, workplace*	Pages 7-8 and in the Abstract
15.	Presence of non-participants	Was anyone else present besides the participants and researchers?	No-one else was present.
16.	Description of sample	What are the important characteristics of the sample? *e.g. demographic data, date*	Pages 9-10
Data collection			
17.	Interview guide	Were questions, prompts, guides provided by the authors? Was it pilot tested?	Use of semi-structure self-report surveys were devised by the research team

No	Item	Guide questions/description	Page numbers where marked in manuscript
18.	Repeat interviews	Were repeat interviews carried out? If yes, how many?	No
19.	Audio/visual recording	Did the research use audio or visual recording to collect the data?	No
20.	Field notes	Were field notes made during and/or after the interview or focus group?	No
21.	Duration	What was the duration of the interviews or focus group?	Page 7 – A 90 minute session of instruction was used with staff and students in preparation for completion of the survey.
22.	Data saturation	Was data saturation discussed?	No
23.	Transcripts returned	Were transcripts returned to participants for comment and/or correction?	Not applicable
**Domain 3: analysis and findings**			
Data analysis			
24.	Number of data coders	How many data coders coded the data?	Two see page 8
25.	Description of the coding tree	Did authors provide a description of the coding tree?	No
26.	Derivation of themes	Were themes identified in advance or derived from the data?	Themes were not derived in advance but from the data.
27.	Software	What software, if applicable, was used to manage the data?	Page 8 see sub-heading 'Data Analysis'
28.	Participant checking	Did participants provide feedback on the findings?	Participants were part of the

No	Item	Guide questions/description	Page numbers where marked in manuscript
			consultation process and provided feedback on the adapted package see stage four of the project.
Reporting			
29.	Quotations presented	Were participant quotations presented to illustrate the themes / findings? Was each quotation identified? e.*g. participant number*	Yes, quotations of text are used to illustrate examples of the themes
30.	Data and findings consistent	Was there consistency between the data presented and the findings?	Yes
31.	Clarity of major themes	Were major themes clearly presented in the findings?	Table 2; see also within the text on pages 9-10
32.	Clarity of minor themes	Is there a description of diverse cases or discussion of minor themes?	No, diverse cases or discussion of minor themes were reported.

For peer review only - http://bmjopen.bmj.com/site/about/guidelines.xhtml

BMJ Open

Cultural adaptation of a UK evidence-based problem-solving intervention to support Polish prisoners at risk of suicidal behaviour: A cross-sectional survey using an Ecological Validity Model.

Journal:	*BMJ Open*
Manuscript ID	bmjopen-2022-069252.R2
Article Type:	Original research
Date Submitted by the Author:	20-Jun-2023
Complete List of Authors:	Perry, Amanda; University of York,<br>Zawadzka, Maja; The Academy of Justice<br>Lapinski, Piotr; University of Physical Education in Warsaw, Faculty of Rehabilitation, Department of Occupational Therapy.<br>Moore, Keeley; Ministry of Justice, HMPPS Stocken<br>Rychlik, Jaroslaw; The Academy of Justice<br>Nowak, Beata; The Academy of Justice
**Primary Subject Heading**:	Mental health
Secondary Subject Heading:	Evidence based practice, Global health, Legal and forensic medicine, Patient-centred medicine
Keywords:	MENTAL HEALTH, Suicide & self-harm < PSYCHIATRY, PUBLIC HEALTH

SCHOLARONE™ Manuscripts

I, the Submitting Author has the right to grant and does grant on behalf of all authors of the Work (as defined in the below author licence), an exclusive licence and/or a non-exclusive licence for contributions from authors who are: i) UK Crown employees; ii) where BMJ has agreed a CC-BY licence shall apply, and/or iii) in accordance with the terms applicable for US Federal Government officers or employees acting as part of their official duties; on a worldwide, perpetual, irrevocable, royalty-free basis to BMJ Publishing Group Ltd ("BMJ") its licensees and where the relevant Journal is co-owned by BMJ to the co-owners of the Journal, to publish the Work in this journal and any other BMJ products and to exploit all rights, as set out in our licence.

The Submitting Author accepts and understands that any supply made under these terms is made by BMJ to the Submitting Author unless you are acting as an employee on behalf of your employer or a postgraduate student of an affiliated institution which is paying any applicable article publishing charge ("APC") for Open Access articles. Where the Submitting Author wishes to make the Work available on an Open Access basis (and intends to pay the relevant APC), the terms of reuse of such Open Access shall be governed by a Creative Commons licence – details of these licences and which Creative Commons licence will apply to this Work are set out in our licence referred to above.

Other than as permitted in any relevant BMJ Author's Self Archiving Policies, I confirm this Work has not been accepted for publication elsewhere, is not being considered for publication elsewhere and does not duplicate material already published. I confirm all authors consent to publication of this Work and authorise the granting of this licence.

For peer review only

Cultural adaptation: problem-solving in prisons

Cultural adaptation of a UK evidence-based problem-solving intervention to support Polish prisoners at risk of suicidal behaviour: A cross-sectional survey using an Ecological Validity Model

Authors:

Amanda E. Perry*

Maja Zawadzka[1]

PiotrLapinski[3]

KeeleyMoore[2]

JaroslawRychlik[1]

Beata Maria Nowak[1]

(with international collaboration from the group of polish prison officers: Kamila Nowak, Anna Szwedowska, Irmina Chrzanowska, Krzysztof Kasjan, Agnieszka Nowogrodzka, Izabela Stankiewicz, Przemysław Worobiej, Anna Kondraciuk, Bartosz Jaroszewski and Sebastian Lizyńczyk).

*Corresponding author

*University of York

Health Sciences Department

2nd Floor ARRC Building

Heslington

York

YO10 5DD

UK

Email: amanda.perry@york.ac.uk

Telephone: +44(0)7795315045

1.The Academy of Justice, Warsaw, Poland.

For peer review only - http://bmjopen.bmj.com/site/about/guidelines.xhtml

2.HMPPS: HMP Stocken, Rutland, UK.

3. University of Physical Education in Warsaw, Faculty of Rehabilitation, Department of Occupational Therapy.

KEYWORDS: Cultural, implementation, problem-solving, suicidal and prisons

WORD COUNT 4173

For peer review only

For peer review only - http://bmjopen.bmj.com/site/about/guidelines.xhtml

ABSTRACT

Objective: To complete a cultural adaptation of a UK evidence-based problem-solving intervention to support Polish prisoners at risk of suicidal behaviour.

Design: A cross-sectional survey participatory design using an Ecological Validity Model.

Setting: The study was a collaboration between: The Academy of Justice, in Warsaw, the University of Lodz, two Polish prisons (ZK Raciborz and ZK Klodzko), and the University of York (UK).

Methods: The adaptation process included an examination of the use of language, metaphors, and content (i.e., culturally appropriate, and syntonic language), the changing of case study scenarios (relevance and acceptability) and maintenance of the theoretical underpinning of the problem-solving model (intervention comprehensibility and completeness). Four stages using: (i) a targeted demonstration for Polish prison staff, (ii) a wider audit of the skills with Polish prison staff and students, (iii) forward and back translation of the adapted package and (iv) two iterative consultations with participants from stages (i) and (ii) and prison officers from two Polish prisons.

Participants: Self-selecting volunteer participants included, targeted prison staff (n=10), prison staff from the wider Polish penitentiary system (n=39), students from the University of Lodz (n=28), and prison officers from two Polish prisons (n=12).

Main outcomes and measures: Acceptability and feasibility of the training package, reported in a series of knowledge user surveys.

Results: The recognised benefits of using the skills within the training package included: enhancing communication, reflective development, collaborative working, changing behaviour, empowering decision making, use in crisis management situations and use of open-ended questions. The skills were endorsed to be used as part of future penitentiary training for prison officers in Poland.

Conclusions: The skills had widespread appeal for use across the Polish penitentiary system. The materials were deemed relevant whilst adhering to the comprehensibility of the

For peer review only - http://bmjopen.bmj.com/site/about/guidelines.xhtml

intervention. Further evaluation of the intervention should be explored using a randomized controlled trial design.

STRENGTHS AND LIMITATIONS OF THIS STUDY

- *Cultural adaptations of prison interventions are rare.*
- *An Ecological Validity Model was used to adapt a UK problem-solving intervention for use within Polish prisons.*
- *The study used small volunteer self-selecting groups of prison staff, students who were training to be prison officers and prison officers in two Polish prisons.*
- *Future engagement with prisoners is key to understanding more about the acceptability and feasibility of the intervention delivery.*
- *A randomised controlled trial is needed to measure the effectiveness of the adapted problem-solving intervention.*

INTRODUCTION

The mental health of people incarcerated in prison is recognized as an international public health concern[1,2]. Prisoners experience disproportionate levels of mental health, isolation, boredom, self-harm, and anti-social violent behaviour when compared to the general population [2-5]. Additionally, prisoners are more than five times more likely to suffer from a common mental health disorder (such as depression) and once diagnosed are more likely to reoffend [6,7] accompanied by the rising demand for health care often outstripping the available resources[8] and continued reports from UK and European prisons of rising incidents of violence assaults, suicidal and self-harm behaviour[9].

International evidence on the effectiveness of interventions for suicidal people in custody (e.g., multicomponent programs, group-based programmes, and peer-support interventions) show many gaps in knowledge and limited use of high-quality research designs such as randomised controlled trials[10]. Reviews of risk factors relating to suicide and self-harm behaviour suggest that interventions should encourage social

For peer review only - http://bmjopen.bmj.com/site/about/guidelines.xhtml

structures, develop positive relationships, and provide purposeful activity by encouraging interventions that utilize a holistic approach to supporting the mental health of prisoners[3,11]. Despite the international recognition of the disproportionate rates of suicide and self-harm behaviour in prisons, there is little evidence to demonstrate the sharing of best practice. Often, interventions devised from community populations are not relevant without further adaptations to the prison environment[12,13,14,15]. This is particularly relevant given that poor adherence (i.e., the degree to which a participant follows the recommendations of an intervention) and rates of attrition are known primary barriers to achieving optimal treatment outcomes for mental health worldwide[16,17]. Interpretation of this data is, in part also about understanding some of the cultural differences between countries in practices and policy.

For example, Poland and the UK have similar prison populations and official statistics of the Polish prison system show rates of suicide, self-harm and aggressive behaviour (https://sw.gov.pl/strona/Statystyka). Although rates of suicide and self-harm behaviour in Poland are relatively low; (between 2015-2019 representing 2.53-3.38 per 10,000 prisoners)[18], cultural differences between Poland and the UK reflect different policies for the recording of self-harm behaviour. In Poland, in 2013 changes to the legal provision of recording self-harm was restricted to the inclusion of only those incidents where cause to harm lasted **more** than seven days. Current self-harm rates recorded in Poland are therefore likely to be significantly less than the actual number of incidents. In addition, stigma associated with reporting mental health problems in Poland has been noted as a barrier to prisoners raising concerns about mental ill health[19].

For many years, both the UK prison service and the Polish penitentiary service have used cognitive behavioural programmes to address offending behaviour [20,21,22]. One element of such programmes includes the use of social problem-solving. In community studies, meta-analytical reviews of problem-solving skills show beneficial impact of reducing symptoms of suicidal ideation (MD -1.58, CI 95% -1.58 to -0.44), outcomes of repetition of self-harm at 4 months (OR 0.65, CI 95% 0.36 to 1.16) and final follow-up (OR 0.76, CI 95% 0.55 to 1.05)[23]. Theorists[24] note that individuals with a characteristic set of negative thoughts and feelings about problems tend to blame themselves for problems and

For peer review only - http://bmjopen.bmj.com/site/about/guidelines.xhtml

doubt their own ability to solve problems effectively. Avoidant and impulsive responses produce problem-solving and thus risk reinforcing negative beliefs and feelings that impact on mental health and well-being[25]. Use of problem-solving skills are important for many who display symptoms of depression, self-harm, or violent behaviour because they often cite a problem as the main cause of their behaviour[26]. In addition, the intervention itself is relatively brief and can be delivered by anyone who is trained to use the skills. As such, the intervention has the potential to be utilised by many in custody.

Prior work in the UK sought to first adapt a community-based seven-step problem solving model producing a co-produced manualised problem-solving training package (consisting of a workbook, digital materials, and handbook) for prison staff and prisoners[27]. The resulting package comprised of five 3-hour group training sessions targeting the following skills (e.g., supporting alternative styles of thinking; self-control; use of social perspective taking and consideration of consequences arising from problem-solving deficits). Key findings from the research evaluation demonstrated a promising reduction in repeat self-harm behaviour[23]. However, problem-solving skills in Poland are not used with those who present with self-harm behaviour or concerns of mental health and well-being. The study therefore aimed to adapt the UK problem-solving model using the Ecological Validity Model process with four stages: first, a demonstration of the training materials to prison staff. This included an evaluation of the feasibility (whether something should be done, should we proceed with it and if so how) and acceptability (how well an intervention has been received by the target population). Second, a wider audit of the skills to gather feedback from the Polish prison community, followed by adaptation of the current training materials using culturally specific examples, use of forward and back-translation, and fourth, two rounds of consultations to identify further amendments to the materials.

METHODS

Overall study collaboration

For peer review only - http://bmjopen.bmj.com/site/about/guidelines.xhtml

The study was conducted using a collaboration between: The Academy of Justice, the University of Lodz, two Polish prisons (ZK Raciborz and ZK Klodzko), in Poland and the University of York (UK). Together the two prisons hold a maximum of 1,474 adult male prisoners. Each prison hosts a range of different functions for short term prisoners and those serving life imprisonment. Including a therapeutic wing for prisoners with diagnoses mental health problems. The Academy of Justice convened the group of prison staff; identified participants for the wider audit; and liaised with prison staff at each prison site. One member of the research team (MZ) held a joint position with the University of Lodz. MZ (a CBT psychotherapist in Poland with knowledge of the scheme) was trained by the PI (AP) in the use of the intervention. The Forensic Mental Health Group and PI (AP) https://www.york.ac.uk/healthsciences/research/mental-health/ourresearch/forensic-mental-health/ from the University of York provided the original materials and was the project lead.

The Ecological Validity Process

An adapted Ecological Validity Model (EVM) [28] included an examination of three different elements; use 'language' and 'metaphors', 'content' reflecting relevance and acceptability and 'concept' to maintain the underpinning theoretical problem-solving model to retain the intervention comprehensibility and completeness. Language is often cited as the carrier of the culture because it presumes ease of delivery, greater familiarity with cultural knowledge, and expressed emotions. As a result, culturally sensitive language maybe instrumental in ensuring that the intervention is well received. Case study scenarios (originally co-produced with people in custody in the UK) were changed to reflect relevant 'content'. They were adapted to provide cultural information about values, customs and traditions that reflect the familiarity of the Polish prison system and the culture of people in Poland (Figure 1).

[INSERT FIGURE 1 HERE]

Knowledge user surveys

For peer review only - http://bmjopen.bmj.com/site/about/guidelines.xhtml

Three separate surveys were devised for stages 1, 2 and 4 of the process. In November 2019 survey one addressed the acceptability of the training and was evaluated using a brief [11-item] self-report questionnaire. The questionnaire was aimed at identifying whether the skills were acceptable and feasible to Polish prison officers. The questionnaire elicited a series of yes/no responses and semi-structured qualitative comments. During January and February 2020 survey two [containing 6-items] was used to gather information on the use of wider use of problem-solving skills with Polish criminal justice staff. Between May 2020 and August 2021 surveys three [containing 8-items] and four [containing 12 items] were used to produce the final iterations of the training package.

PROCEDURE

Stage one: Demonstration of the training package for Polish prison staff.

The problem-solving demonstration sessions were delivered by KM and AP in The Academy of Justice during a three-day period in November 2019. Ten prison staff members were invited by The Director of the Academy of Justice. Apart from having a willingness to participate, staff had to meet two further criteria: (i) to have good knowledge of English, and (ii) to work in a position where the skills could be used in the future. The sessions included a demonstration of the problem-solving skills, sharing of the UK case study scenario examples and a series of role play exercises to support the adaptation of the materials (see supplementary materials). At the end of the training session, staff were asked to provide feedback using survey one.

Stage two: Audit of the problem-solving skills with staff working in the wider Polish prison community.

During January and February 2020, the audit was used to gain a wider perspective from a larger population of prison staff and students who were training to become a prison officer. MZ individually self-selected and approached staff and students to take part in the study. MZ

For peer review only - http://bmjopen.bmj.com/site/about/guidelines.xhtml

demonstrated the problem-solving skills using a brief 90-minute session using a five-minute animation and delivery of the problem-solving skills. Staff and students were showed copies of the training workbooks before completion of survey two.

Stage three: Adaptation of the materials into Polish

Feedback from stages one and two informed the translation of the training documents. The translation from English into Polish was completed by MZ. Back translation was completed by an independent translation service in the UK. In addition, the translation was checked against the original text by the project PI (AP). Ratification of the text was completed with further discussion with colleagues in Poland to ensure the wording and language was comparable and culturally appropriate[29].

Stage four: Consultation process of the adapted materials

Feedback from staff already engaged in stages one and two were asked to take part in two rounds of consultation. Additionally, in round two, 12 prison officers were recruited from two Polish prisons to review the training package. During the approach by MZ, 10 prison officers declined to take part due to the 'pressures of daily duties'. The findings from the consultation process were categorised into changes relating to the EVM model including language, metaphor and content, (relevance and acceptability) and content (intervention comprehensibility and completeness).

Data Analysis

All survey results were anonymized, and data were entered into a series of different Excel spreadsheets. IBM SPSS Statistics (version 26) and Statistica StatSOFT version 13 were used to explore the quantitative responses generating mean scores and descriptive statistical information. The distribution of the data was examined to identify use of either parametric or nonparametric testing at ($p<0.05$). The qualitative comments were summarized at each stage in the study, and input into Excel software. Inductive coding was used to synthesise the evidence and then generate themes[30]. The COREQ checklist (http://www.equator-network.org/reporting-guidelines/coreq/) was completed to ensure transparency of the methodology. Coding was completed by MZ and AP generating themes. Qualitative comments within each theme were

For peer review only - http://bmjopen.bmj.com/site/about/guidelines.xhtml

additionally 'counted' to provide a numerical frequency which enabled a direct comparison of comments within each theme. The responses from stage two were divided into those reporting either by Prison Staff (PS) or students (S) in training.

Patient and public involvement

The PPI group were involved in the design of the adapted workbooks through the series of consultations. The feedback from this engagement determined the final product.

RESULTS

Stage one: Demonstration of the training package for Polish prison staff

Characteristics of the training sample

Prison staff (n=10) were aged between 29 and 39 years of age (mean age 34.7 years, SD = 3.25). All identified as white European and spoke Polish as their first language. Most of the group were female 7/10 (70%); one participant considered themselves disabled. Participants had worked in the prison service for an average of 103.4 months (SD = 39.8, min = 51 months, max = 178 months), and within the current prison for a mean number of 65.7 months (SD = 35.5, min = 6, max = 111). Prison staff had different roles, including psychologists, penitentiary departmental managers (providing substantive supervision over psychologists and educators) and specialists from the Central Board of the Prison Service. All staff reported dealing with an incident of self-harm or attempted suicide within an average of 16 months prior (SD = 15.8, min = 1 month, max = 36 months). Of those reported incidents 1/10 (10%), reported self-poisoning, 3/10 (30%) reported general self-injury, 4/10 (40%) reported attempted suicide. Incidence of violence was reported by 7/10 (70%) and 3/10 (30%) reported an incident that could have been avoided. Most prison staff 7/10 (70%) encountered prisoners with mental health problems on a daily or weekly basis 2/10 (20%).

Acceptability of the training package and use of the skills

For peer review only - http://bmjopen.bmj.com/site/about/guidelines.xhtml

All participants thought that the training was useful and most learned new skills (Table 1). Five main themes included: an opportunity to learn new skills ('new knowledge' and a 'different approach is always useful'), providing useful communication skills ('learning how to speak to prisoners'), developing reflection and use of perspective taking ('to view it through the prisoner's perspective'), use of a collaborative approach ('working towards a joint search for a solution, not imposing one views'), and use of open-ended questions to progress behaviour change ('work on changes in thinking is important'). Most staff felt that training could be delivered by prisoners, prison staff, psychologists, educators, and practitioners within the prison site. Half the group wanted the training to be longer and nearly all thought that an additional booster session would be useful.

Table 1: Results following demonstration of the training package for Polish prison staff.

Questionnaire	n/10 (% yes)	Qualitative comments
In what ways was the training helpful to you?	NA	Development of a new perspective<br>A new technique/tool kit/ alternative approach<br>Exchange of ideas between the UK & Poland
What are the positive aspects of the scheme?	NA	Straight forward, simple, easy to understand.<br>Problems solved by prisoners.<br>The development of coping skills<br>Group work<br>Providing a structure to help change
Is the training applicable for your job role?	10/10 (100%)	NA
Did you learn any new skills?	7/10 (70%)	NA
If yes, what new skills did you learn?	NA	Working from the prisoner's perspective<br>How to speak to prisoners<br>Development of problem-solving skills

For peer review only - http://bmjopen.bmj.com/site/about/guidelines.xhtml

Who do you think is best to offer the training to prison staff or prisoners?	NA	Prisoners, prison staff, psychologists, educators, practitioners within the prison site.
Is the training relevant to your job role in the prison?	9/10 (90%)	NA
What improvements could be made to the scheme?	NA	Development of more practice exercises<br>Use of additional case study examples<br>Development of the materials
Is the length of the training adequate?	5/10 (50%)	The training should be longer.
Would it be useful to have a booster session?	9/10 (90%)	NA
How will the training be sustained?	NA	In everyday use<br>Training of prisoners on an individual basis<br>Training as part of prisoner officer rules

Stage two: Audit of the problem-solving skills with staff working in the wider Polish prison community.

Characteristics of the audit sample

67 volunteer participants took part in the wider audit including: 39/67 (58%) prison staff and 28/67 (42%) students. Prison staff including 30/39 (77%) males with ages ranging from 20 to 50 years of age. Just under a third, 12/39 (31%) worked as Educators, 3/39, (7%) were Prison Management, 15/39 (38%) worked in the Security Department, 10/39 (25%) were Shift Commanders; 1/39 (2%) worked in the Finance Department, 2/39 (5%) worked in the Quartermaster Department, 3/39 (7.8%) worked in the Therapeutic Department; and 2/39 (5%) were prison Inspectors. Students were aged between 20 and 30 years of age and just under a third 9/28 (32%) were male. All students were enrolled on a Master's degree in National Security and upon completion would join the Polish prison service.

Overall results from the audit

Table 2 shows the overall findings from the audit. Both prison staff and students thought that the

For peer review only - http://bmjopen.bmj.com/site/about/guidelines.xhtml

problem-solving skills should be taught as part of standard training. The perceived benefits were wide ranging. Prison staff were more likely than students to recognise use of the skills as an opportunity to change behaviour, to empower others to increase the pace of decision making 28/67 (42%), develop knowledge 30/67 (45%) and apply the skills in instances of crisis management 18/67 (27%). Students reported use of the skills in general problem solving 22/67 (33%). Staff were more likely that students (37% vs 11%) to use the skills for self-development and knowledge. Students were more likely than staff (35% vs 15%) to use the skills to develop their own coping strategies. Over half of the prison staff 23/39 (58%) and most of the students 19/28 (70%) saw relevance of the skills within their job role. No significant differences were found in the opinion of students or staff in the perceived beneficial of integrating the skills into the training scheme for newly qualified prison officers ($X^2(1, N = 66) = 3.45, p > 05.$). Prison staff when compared to students were significantly more likely to accept that the training could be translated into skills for prisoners **(**U=51, median rank: 243; 222), p<0.05.

Table 2: Audit of the acceptability of the problem-solving skills with a wider Polish prison community

Questionnaire items	PO (N% yes)	Student (N% yes)	Prison Officer (PO) and Student (S) Qualitative themes	
Do you think problem-solving skills should be taught to prison officers as part of standard training?	38/39 (97%)	23/27 (85%)	NA	NA
Do you think there are benefits from learning the skills?	34/39 (87%)	24/27 (88%)	NA	NA
If yes, what are the benefits?	NA	NA	Changing behaviour (PO=9/33 27% vs S=2/24 8%)<br>Crisis planning & prevention (PO=2/33 6% vs S=3/24 12%)<br>Developing coping strategies (PO=2/33 6% vs S=3/24 12%)<br>Developing personal skills (PO=1/33 3% vs S=1/24 4%)	

For peer review only - http://bmjopen.bmj.com/site/about/guidelines.xhtml

			Developing social networks (PO=4/33 12% vs S=1/24 4%)<br>Generating solutions (PO=1/33 3% vs S 1/24 4%)<br>Helping someone (PO=0/33 0% vs 5/24 20%)<br>General problem solving (PO=2/33 6% vs S=8/24 33%)<br>Empowerment to make decisions at pace (PO=14/33 42% vs 2/24 8%).	
What do you like most about the idea of using the skills?	NA	NA	Ability to compromise (PO=3/32 9% vs S=0/17 0%)<br>Knowledge & self-development (PO=12/32 37% vs S=2/17 11%)<br>Developing coping strategies (PO=5/32 15% vs S=6/17 35%)<br>Empowerment (PO=6/32 18% vs S=5/17 29%)<br>Tailored to individual need (PO=1/32 3% vs S=0/17 0%)<br>Improved communication (PO=1/32 3% vs S=2/17 11%)<br>Accessible to help others (PO=4/32 12% vs S=2/17 11%)	
Could you see them being used in your job role?	23/39 (58%)	19/27 (70%)	NA	NA
If yes, in what capacity?	NA	NA	Solving problems (PO=1/22 4% vs S=3/16 18%)<br>Helping others (PO=0/22 0% vs S=3/16 18%)<br>Applicability in life experiences (PO=0/22 0% vs S=2/16 12%)<br>Developing empathy (PO=0/22 0% vs S=2/16 12%)<br>Crisis management (PO=6/22 27% vs S=4/16 25%)<br>Increased knowledge (PO=10/22 45% vs S=1/16 6%)<br>Empowerment (PO=3/22 13% vs S=1/16 6%)<br>Social cultural activities (PO=2/22 4% vs S=0/16 0%)	

Stage three: Adaptation of the materials into Polish

Feedback from stages one and two enabled the materials to be translated into Polish using a series of changes within the structure of the EVM. These included:

For peer review only - http://bmjopen.bmj.com/site/about/guidelines.xhtml

Language, metaphors, content

Words were added to the workbooks (e.g., 'to be' or 'to have') to ensure that the context of the prison environment were more understandable in the Polish language. Words were also changed to reflect different cultural differences. In addition, some English words were non-existent in Polish and in these cases similar words were found and replaced to ensure that the comprehension of the materials remained the same.

Relevance and acceptability

One male case study scenario was reviewed as part of the training package (supplementary materials). Changes to the case study scenario ensured its applicability to the Polish penitentiary system. Such changes included the consideration of the daily routine of prisoners and the signposting of available resources in Poland (which were inevitably different to those in the UK). Options generated from step four of the problem-solving model that did not exist within the Polish context were changed to reflect options that would be acceptable to Polish prisoners.

Intervention comprehensibility and completeness

The workbook titles were changed to reflect the comments made during stages 1 and 2. The theoretical underpinning of the intervention was adhered to throughout the structure of the workbooks to ensure that the intervention comprehensibility was maintained. British names of resources were changed to Polish resources to reflect the completeness of the intervention.

Stage four: Consultation process of the adapted materials

Characteristics of the sample: first and second consultations

Two rounds of consultation included in round one: (i) prison staff from stage one 8/10 (80%), and a (ii) wider group of professionals 10/39 (25%) from stage two. Round one results showed that most people (83%) liked or really like the pictures and diagrams, 89% reported both that the language reflected the context of the prison environment; and the skills were perceived as useful within daily work. 100% of participants reported that they would like to attend training.

For peer review only - http://bmjopen.bmj.com/site/about/guidelines.xhtml

Round two included those participants who took part in round one plus a group of serving prisoner officers (n=12) from two Polish prisons who had not previously seen the materials (Table 3).

Table 3: Consultation process of adapted materials round two

Second consultation (n=28)	Definitely not (yes)	Rather not (yes)	No strong preference	Rather (yes)	Definitely (yes)
Quality	0	1 (4%)	2 (7%)	15 (53%)	10 (36%)
Clarity	0	0	5 (18%)	12 (43%)	11 (39%)
Diagrams	0	1 (4%)	2 (7%)	13 (46%)	13 (46%)
Structure	0	2 (7%)	2 (7%)	15 53%)	8 (29%)
Materials	0	0	4 (14%)	17 (60%)	6 (21%)
Training modules	0	2 (7%)	2 (7%)	14 (50%)	9 (32%)
Understanding clarity and content	0	1(4%)	5 (18%)	14 (50%)	7 (25%)
Acceptability of the case study	1 (4%)	1 (4%)	2 (7%)	15 53%)	8 (29%)
Language adaptation	1 (4%)	1 (4%)	2 (7%)	14 (50%)	10 (36%)
Possibility of self-development	1 (4%)	1 (4%)	5 (18%)	18 (64%)	3 (11%)
Development of coping strategies	0	2 (7%)	3 (11%)	14 (50%)	8 (29%)
Level of innovation: use within the system	0	1 (4%)	3 (11%)	14 (50%)	9 (32%)

Language, metaphors, content

The combined results from the qualitative consultations in rounds one and two showed that overall, the language used were appropriate for the prison context ('I rate the materials at a level appropriate to the subjects' language. 'I think it is understandable and also simple'). The second consultation identified some useful further iteration on use of appropriate language to

For peer review only - http://bmjopen.bmj.com/site/about/guidelines.xhtml

('unify terminology' and reflected that 'some words are not good in non-changed from original version form'). Based on this feedback a final edit of the materials was completed.

Relevance and acceptability

The workbooks contained diagrams and pictorial representations through cartoon like characters. These were used in the original UK workbooks to aid literacy levels[31,32]. In Poland, most prison staff thought these were acceptable with wider benefit to other client groups recognized ('the presented training materials are interesting their great value is universalism'; they can be implemented with different groups of people'). The general opinions about the overall presentation of the workbooks were good ('diagram and pictures facilitate understanding of the presented content').

DISCUSSION

To our knowledge this study is the first to demonstrate use of the EVM to develop the cultural adaptation of an evidenced-based UK problem solving skills training package for prison staff and prisoners at risk of self-harm, mental health, and well-being in Poland. Cultural adaptation procedures are key in the acceptability and recognized feasibility of using any intervention[12]. Iterative input from Polish prison staff was vital in generating a training package with the potential to support good adherence. The guiding principle of cultural adaptation is to work closely with stakeholders during early developmental stages to ensure that they can be sustained [32-33].

The results demonstrated acceptance of the skills regardless of staff or student group, indicating that the use of the skills had potential widespread appeal across the Polish penitentiary system. Similar findings were found in the prior UK studies with high levels of acceptability and relevance reported by prison staff and prisoners[24]. As expected, staff in comparison to students could envisage greater application of the skills within the prison context. This may well reflect the experience and knowledge of prison staff who were already working within the Polish prisons. Prisons are challenging settings to implement the

For peer review only - http://bmjopen.bmj.com/site/about/guidelines.xhtml

development of new skills whereby the nature of the environment presents complex issues of trust, confidentiality, and fairness with arguably many logistical and practical barriers to implementation[34]. Failure to generate interventions without high levels of acceptability or feasibility is a barrier to wide-spread use of interventions that may ultimately bring benefit to reductions in self-harm mental health and well-being. Interventions that engage staff from across the prison sites help consider the integration and development of sustainable models to prevent self-harm behaviour and promote mental health and well-being going forward.

Limitations

The study has several limitations. First, the volunteer self-selecting nature of the sampling is unlikely to reflect the representative nature of all staff in the Polish prison service. Arguably, the nature of the sampling within this evaluation may impact on the generalisability of the training package. Extensions of this work would require feedback from a diverse range of prisoners (including females) and use of novel participatory strategies. Although feedback from staff reported that the skills would be relevant in circumstances of 'crisis management'; further evaluation of the training package should gather views on the perceived impact on the management of suicidal and self-harm behaviour.

Implications and future research

Our adaptation processes resulted in good levels of acceptability and feasibility, with positive feedback from prison staff and students. Recommendations refer to the necessity of using problem-solving skills as a part of standard training, by integrating skills such as role-play to support communication skills. Further development of the workbook and training package need to consider further adaptations that will help to ensure that the materials are widely accessible to prisoners with different protected characteristics. The inclusion of further narrative case studies for females in prison would require additional consideration.

CONCLUSION

For peer review only - http://bmjopen.bmj.com/site/about/guidelines.xhtml

The process of cultural adaptation enables researchers to efficiently tailor evidence-informed interventions for diverse populations while retaining the core components that underpin efficacy. The EVM provides a useful framework for adapting interventions. Use of this method produced an acceptable training package for the Polish prison service. Further evaluation of the effectiveness of the problem-solving training skills needs to be explored using a randomised controlled trial design.

DATA AVAILABILITY STATEMENT

Data is made available upon reasonable request.

ETHICS STATEMENTS

Ethical approval for the study was granted by the Department of Health Sciences at the University of York in the UK and reviewed by a RODO Data Protection Specialist in The Academy of Justice, in Warsaw, Poland. Permission was granted from each prison in taking part in the study. All study participants received full informed consent.

ACKNOWLEDGEMENTS

With many thanks to the UK HMPPS Intervention services, to all the prison staff that contributed to the project including the prisons of ZK Racibórz and ZK Kłodzko. To the Central Board of Prisons in Poland who gave permission for this research.

FUNDING

This study was funded through the Wellcome Trust, Centre for Future Health, Rapid Response Fund, University of York, UK.

AUTHOR CONTRIBUTION STATEMENT

AP and MZ devised the study with the initial support from PL. AP and KM delivered the training in stage one. JR conducted the analysis and BNM supported Polish colleagues in the written work for the publication. All elements of the project were overseen by AP. All authors worked on the draft of the manuscript.

COMPETING INTERESTS

None declared.

For peer review only - http://bmjopen.bmj.com/site/about/guidelines.xhtml

FIGURE LEGENDS

Figure 1 Overview of study process

REFERENCES

1. Durcan, G. (2021). The future of prison mental health care in England. M Centre for mental health. [CentreforMentalHealth_TheFutureofPrisonMentalHealthCare_0.pdf]. Accessed September 2022.

2. Fazel, S., Hayes, A. J., Bartellas, K., Clerici, M., & Trestman, R. (2016). Mental health of prisoners: prevalence, adverse outcomes, and interventions. Lancet Psychiatry, 3, 871-881 https//doi: 810.1016/S2215-0366(1016)30142-30140. Epub 32016 Jul 30114. PMID: 27426440; PMCID: PMC25008459.

3. Favril, L., Yu, R., Hawton, K., & Fazel, S. (2020). Risk factors for self-harm in prison: a systematic review and meta-analysis of 35 studies covering 663735 adults. Lancet Psychiatry, 7(8), 682-691. https//doi: 610.1016/S2215-0366(1020)30190-30195.

4. Brugha, T., Singleton N, Meltzer H, Bebbington P, Farrell M, & Jenkins R, G., L. (2005). Psychosis in the community and in prisons: a report from the British National Survey of psychiatric morbidity. American Journal of Psychiatry, Apr;162(4):774-80. https//doi: 10.1176/appi.ajp.162.4.774. PMID: 15800152. 774-780.

5. Favril, L., Indig, D., Gear, C. *et al.* (2020) Mental disorders and risk of suicide attempt in prisoners. *Soc Psychiatry Psychiatr Epidemiol* **55**, 1145–1155. https://doi.org/10.1007/s00127-020-01851.

6. Favril, L., Shaw, J., Fazel, S. (2022). Prevalence and risk factors for suicide attempts in prison, *Clinical Psychology Review, Volume 97, https://doi.org/10.1016/j.cpr.2022.102190*.

7 Favril, L., O'Connor, R., Hawton, K., & Vander Laenen, F. (2020). Factors associated with the transition from suicidal ideation to suicide attempt in prison. *European Psychiatry, 63*(1), E101. https//doi:10.1192/j.eurpsy.2020.101.

8. Vos, T., Lim, S. S., Abbafati, C., Abbasi, M., & Abbasi-Kangevan, M. e. a. (2020). Global burden of 369 diseases and injuries in 204 countries and territories, 1990-2019: a systematic review analysis of the Global

For peer review only - http://bmjopen.bmj.com/site/about/guidelines.xhtml

Burden of Disease Study 2019. The Lancet, 396(10258: 1204-1222: doi), 1204-1222: https//doi.org/1210.1016/SO1140-6736 (1220) 30925-30929.

9. Ministry of Justice 2022 Safety in Custody Statistics, England and Wales: Deaths in Prison Custody to December 2017 Retrieved from National Statistics: Ministry of Justice, 2022: https://www.gov.uk/ /collections/safety-in-custody-statistics. Accessed April 2023.

10. Carter, A. Butler, A. Willoughby, M. Janca, E., Kinner, S.A., Southalan, L., Fazel, S. Borschman, R. Interventions to reduce suicidal thoughts and behaviours among people in contact with the criminal justice system: A global systematic review. *E ClinicalMedicine 2022;44: 101266 Published online 14 January 2022 https://doi.org/10.1016/j. eclinm.2021.101266*.

11. Perry, A. E., (2020). Self-harm in prisons: what do we know and how can we move forwards? www.thelancet.com/psychiatry Vol 7 August 2020, 649-650. https://doi.org/10.1016/S2215-0366(20)30298-4.

12. Culbert, G.J. & Williams, A. B. (2018). Cultural Adaptation of a Medication Adherence Intervention With Prisoners Living With HIV in Indonesia: A Pragmatic Approach to Intervention Development. Journal of the Association of Nurses in AIDS Care, 29, Issue 3, Pages 454-465,ISSN 1055-3290, https://doi.org/10.1016/j.jana.2017.11.006.

13. Thakker, J. (2014). Cultural factors in offender treatment: Current approaches in New Zealand. Social and Behavioural Sciences, 113, 213-223. https//DO 10.1016/j.sbspro.2014.01.028.

14. Castro, F. G., Barrera, M. J., & Holleran Steiker, L. K. (2010). Issues and challenges in the design of culturally adapted evidence-based interventions. Annual Rev Clin Psychology 6:213-39. https//doi: 10.1146/annurev-clinpsy-033109-132032.

15. Faregh, N., Lencucha, R., Ventevogel, P., Dubale, B. W., & Kirmayer, L. J. (2019). Considering culture, context and community in mhGAP implementation and training: challenges and recommendations from the field. Int J Ment Health Syst 13:58. https//doi: 10.1186/s13033-019-0312-9.

16. Reingle Gonzalez, J. M., & Connell, N. M. (2014). Mental health of prisoners: identifying

For peer review only - http://bmjopen.bmj.com/site/about/guidelines.xhtml

barriers to mental health treatment and medication continuity. American journal of public health, 104(12), 2328-2333. https://doi.org/10.2105/AJPH.2014.302043.

17. Nagpal, T.S., Mottola, M.F., Barakat, R., Prapavessis, H. (2021). Adherence is a key factor for interpreting the results of exercise interventions, *Physiotherapy, Volume 113, Pages 8-11, https://doi.org/10.1016/j.physio.2021.05.010.*

18. Lizińczyk, S. (2023). Characteristics of suicides committed in Polish prisons, 2015–2019: *Archives of Criminology*, (XLII/2), 313–336. https://doi.org/10.7420/AK2020P

19. Przybyliński S., Marczak M. (2008). Self-mutilation among female and male prisoners. In: W. Ambrozik, H. Machel, P. Stępniak (eds.) The mission of the Prison Service and its tasks in relation to the current penal policy and social expectations. IV Polish Penitentiary Congress (p. 455) Poznań-Gdańsk-Warsaw: UAM, UG, Pedagogium WSPR and CZSW.

20. Gobbett MJ, & Sellen, J.L. (2014). An evaluation of the HM prison service thinking skills programme using psychometric assessments. Int J Offender Therapy Comp Criminol., Apr;58(4):454-73. https//doi: 10.1177/0306624X12472485. Epub 2013 Feb 6. PMID: 23390065.

21. Majcherczyk, A. (2011). Selected rehabilitation programs - theoretical and practical perspective. *Przegląd Więziennictwa Polskiego", nr 70, p. 5-30.*

22. Rejzner, A., & Szczepaniak, P. (2009). Therapy in rehabilitation, volume I Theoretical approach & volume II A practical approach. Warszawa: Żak.

23. Perry, A. E., Waterman, M., Dale, V., Moore, K., & House, A. (2021). The effect of a problem support intervention on self-harm and violence in prison: an interrupted time series analysis using routinely collected prison data. EClinical Medicine- The Lancet, 32 (1007021), 1-11 https://doi.org/10.1016/j.eclinm.2020.100702.

24. D'Zurilla, T. J., Chang, E. C., Nottingham, E. J. IV, & Faccini, L. (1998). Social problem-solving deficits and hopelessness, depression, and suicidal risk in college students and psychiatric inpatients. *Journal of Clinical Psychology, 54*(8), 1091–1107.https://doi.org/10.1002/(SICI)1097-4679(199812)54:8<1091::AID-JCLP9>3.0.CO;2-J.

For peer review only - http://bmjopen.bmj.com/site/about/guidelines.xhtml

25. D'Zurilla, T. J., & Goldfried, M. R. (1971). Problem solving and behavior modification. *Journal of Abnormal Psychology, 78*(1), 107–126.https://doi.org/10.1037/h0031360.

26. Linehan, M. M., Camper, P., Chiles, J. A., Strosahl, K., & Shearin, E. (1987). Interpersonal Problem-Solving and Parasuicide. Cognitive Therapy and Research, 11(1), 1-12. https//Doi 10.1007/Bf01183128.

27. Perry A. E., Waterman MG, House A, *et al* Problem-solving training: assessing the feasibility and acceptability of delivering and evaluating a problem-solving training model for front-line prison staff and prisoners who self-harm *BMJ Open* 2019;**9:**e026095.https//doi: 10.1136/bmjopen-2018-026095.

28. Bernal, G., Bonilla, J., & Bellido, C. (1995). Ecological validity and cultural sensitivity for outcome research: issues for the cultural adaptation and development of psychosocial treatments with Hispanics. J Abnorm Child Psychol 23(1):67-82. https//doi: 10.1007/BF01447045.

29. Ozolins, U., Hale, S., Cheng, S. X., Hyatt, A., & Schofield, P. T. (2020). Translation and back-translation methodology in health research - a critique. Expert Review of Pharmacoeconomics & Outcomes Research, https//DO 10.1080/14737167.2020.1734453.

30. Braun, V., & Clarke, V. (2013). Successful qualitative research: A practical guide for beginners. London: Sage.

31. Morken, F., Jones, L. Ø., & Helland, W. A. (2021). Disorders of Language and Literacy in the Prison Population:A Scoping Review. Educ. Sci., 11, 77. https://doi.org/10.3390/educsci11020077. Najwyższa, I. K. (2020). Security of inmates. Information on the inspection results. KPB.430.002.2020, Nr ewid. 52/2020/P/19/040/KPB, available online: https://www.nik.gov.pl/plik/id,22869,vp,25572.pdf [Accessed January 2022].

32. Castro, F. G., & Yasui, M. (2017). Advances in EBI development for diverse populations: towards a science of intervention adaptation. Prevention Science, 1-7. https// doi: 10.1007/s11121-017-0809-x.

33. Williams, A. B., Wang, H., Burgess, J., & X., L. (2013). Cultural adaptation of an evidence-based nursing intervention to improve medication adherence among people living with

For peer review only - http://bmjopen.bmj.com/site/about/guidelines.xhtml

HIV/AIDS (PLWHA) in China. International Journal of Nursing Studies, 50(4):487-494. https//doi: 10.1016/j.ijnurstu.2012.08.018.

34. Azbel, L., Grishaev, Y., Wickersham, J. A., Chernova, O., Dvoryak, S., Polonsky, M., & Altice, F. L. (2016). Trials and tribulations of conducting bio-behavioral surveys in prisons: implementation science and lessons from Ukraine. International Journal of Prisoner Health, 12(2):78-87. https//doi: 10.1108/IJPH-10-2014-0041. 25245352.

For peer review only

For peer review only - http://bmjopen.bmj.com/site/about/guidelines.xhtml

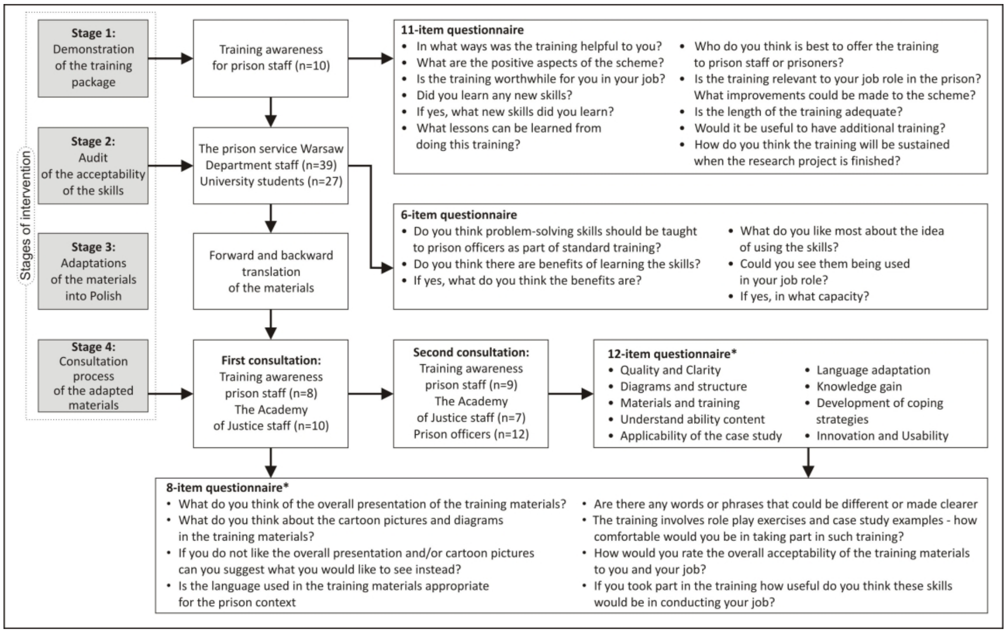

Figure 1: Study Flow Process

263x164mm (300 x 300 DPI)

Supplementary File

Session one: Role play example – demonstrating how a negative attitude can affect the outcome of a problem

- Facilitator introduce the first role-play example to show about people behave and react to a situation can vary depending upon how they feel:

'That you are on your way to an interview in your car and you suddenly realised that you have t wo but two flat tyres. You suspect that a neighbour has let your tyres down. All dressed up in a suit and tie. You have to stop I the middle of the road blocking the traffic which grinds to a halt behind you which starts causing chaos.' A bus stop is near by. Someone in another car stops and laughs at you – pulls over the stops you go over to them…

- Facilitators to demonstrate the scenario

- Facilitator to question the group:
  - How did the driver feel about the situation?
  - What was there attitude like?
  - Did the incident end in a positive way?
  - What could they have done differently/said?

Facilitator to encourage the group to mimic the role-play exercise so…

- Ok so I want you to get into pairs. One person be the driver who approaches the person who has laughed at them. I want you to start your conversation telling the person how you feel what your problem is and see how they respond.
- When we have done this we are going to go around the room and talk about what happened in each person's situation – was the problem resolved- did the person help – did they get to the interview or not?
- Then repeat the exercise and role play it with a positive attitude. The exercise shows that if you do things in the right way you can often get a more satisfactory outcome.

On a flip chart and with the whole group the facilitator uses the example to demonstrate the problem-solving steps.

For peer review only

Steps in the model:

Step1: Getting the right attitude

Step 2:Reflect and recognise triggers – facilitator notes that if people reflect on previous incidents that didn't turn out as anticipated this can help to identify how they felt at the time, what they thought and how they behaved. Recognising these triggers can play an important role in behaviour change.

Step 3: Define the problem clearly – often we don't have all the information we need to resolve a situation and sometimes you might use these skills to help support how someone feels to enable them to cope better with a situation. Facilitator to prompt – what information did the person does not have in the car scenario. Using what/where/when/why type questions can help to elicit this information in more detail. This specific information helps someone to explore the crux of the problem. To use the problem-solving skills effectively a problem needs to be clearly defined. For example, 'I have a problem with my wife' isn't a problem that is sufficiently defined to be able to use the skills.

Step 4 Brainstorming solutions – Once we have as much information as possible and a clear problem definition, we encourage people to think of as many different solutions (good or bad) to help promote ideas that the person might want to try out. In this model of problem solving, it is important that the person comes up with their own ideas (see the list of suggestions in Appendix 1) if someone requires some support to do this.

Step 5 Decision making – For each solution we then make a list with the possible pros and cons of each idea. It is good to go through the 'not so positive ideas' as it allows someone to explore what might go wrong is they choose an option that is less than favourable. This process helps someone to shortlist the ideas to two or three that they wish to pursue and take forwards into an action plan.

Step 6 Make a plan -Then we think about making a plan – this can be a different plan for everybody [the right plan for one person might not be the case for someone else]. Write down your plan – what would you do? What is your plan? The more specific the plan is the more likely it will work. In what order are you going to do the tasks? When are you going to do each task? Do you need some help or support to complete the task?

Step 7 Review – Problem-solving is an iterative process and not always does it work for many logistical reasons. It is therefore sensible to support an individual to review what happened. Did your plan work? What went wrong? Using the skills again to produce another alternative and try again using the skills. The more someone practicing the skills the better they get at using them.

For peer review only

Male Case Study Example:

James is 22 years old. He is one of six siblings and lives with his father and stepmother. James's relationship with his parents was troubled from a young age and his father would come home drunk and beat James. His stepmother found it difficult to deal with James's aggressive emotional outbursts and he was excluded from school at age 11 for poor behaviour and angry outbursts.

James started to mix with a gang of older boys who were known in the area for committing petty crimes. He became involved in drugs at age 13 years and was caught by the police for burglary when he was 16 years old. He also had a series of relationships with older women which led to a number of pregnancies resulting in two sons and a new baby. James's stepmother was unable to control his behaviour and did not want him in the house anymore so James was asked to leave.

James went to stay with a friend but soon ended up living in a hostel. He found it difficult to get a job and ended up stealing to support his drug habit. His physical and mental health deteriorated and he no longer took care of himself. One day he took drugs and alcohol and ended up in the Accident and Emergency department at the hospital.

James was finally convicted for a series of burglaries and ended up in prison for the first time. At an all-time low James has contact with his family, with his partner visiting him with the kids on a regular basis. James regularly self-harmed when he was feeling particularly stressed in prison. James was placed in a shared cell and though having settled into prison life James felt angry and frustrated.

On a recent prison visit his partner told him that the council were planning to change their accommodation because James was no longer living with them. The change in circumstances would mean that James's partner could be moved outside of the local area. James returned to the wing in a low mood. He feels inadequate and powerless to do anything about the change in circumstances. James 'kicks off' in his cell.

Recognising and Identifying Problems

Facilitators encourage the group to use the 7-step model to work through the case study example.

For peer review only

Re-visit case study

James felt daunted by trying out brainstorming. At first he felt that nothing would help the situation. As James and his prison officer started to work together he gained momentum with the situation and provided a number of different ideas which helped him feel more in control of the situation.

'my partner is being forced to move out of her house and is being moved away from the area and I will not see my family every week at visiting time'

Here are some practical solutions

Ask my partner to ring the council and find out where she is moving to

Arrange a specific time when they will be able to visit so I can look forward to the visit

Get some photos of my baby and partner to put up in my cell

Ring my partner more often

Talk to visits to see if I can get extended visiting time when they come

Here are some emotional solutions

Focus on keeping myself to myself and not getting into trouble whilst in prison

Plan the time I have in prison to keep me busy

Ask if I can have extra jobs to do in the prison to keep my mind occupied

Go to the prison gym and take part in some exercise

Consider going to education to see if they have any courses I can attend

For peer review only

Re-visit the case study

When James looked at the initial selection to her brainstorm he decided to cross out. He thought they lacked feasibility and had a negative effect on his wellbeing.

The James grouped the remaining ideas. She grouped them into three categories:

Activities to keep himself busy

Methods of organising time

Strategies to manage stress

After thinking about the advantages and disadvantages of these possibilities James decided that planning time in advance had the advantage of reducing his stress. James solution was first to speak to his personnel officer about contacting education and the jobs section within the prison to find out more about what opportunities were available to him.

James also wanted to ring his partner to discuss the move location and ask her to send some pictures of the baby for his cell wall.

Overall, James thought the combination of these two strategies had a good chance of allowing him to feel better about his time in prison and seeing his family less.

SMART action plan and reviewing progress

SMART – Action plan

**Specific**	clear definition of what the action is
**Measurable**	it can be clearly determined whether or not the action has been achieved
**Achievable**	it is important to break the steps down so they can be successfully completed
**Relevant**	action is linked to the problem
**Time-bound**	timescale by which the action will be achieved

For peer review only

Re-visit the case study

James had to create two action plans one for each solution. They contributed to solving the same problem and could be implemented at the same time.

Extra activities in prison action plan

James decided he would ask his personnel officer about how to approach education and the jobs department in the prison. Once he had done this and gathered some more information James discovered that he needed to complete a jobs request card and place it in the post-box on the wing. He also discovered that he could go to education and speak with one of the teachers directly about what was available. In the next few days James identified the next tasks:

1. He considered his job options and also the potential demand to do such jobs within the prison. He wanted to choose some jobs that he would be allocated quickly, maybe because other people did not want to do them.
2. He decided to speak to his personnel officer again and other prison inmates to try and assess which job options he might have most success with.
3. Once he had gained the information he made a decision to put down a range of job choices with the hope of him being allocated something. These included working in the kitchen and canteen, cleaning on the wings and the toilet areas, working in the workshop making prisoner uniform for other prisons.
4. He was told that he would hear back about his choice of jobs within one week
5. He also planned a visit to education to discuss his course options

For peer review only

Further practice role play examples:

David has been on the wing for 12 months and has become addicted to drugs referred to as SPICE – on the last occasion he only narrowly escaped death and the healthcare staff had to bring him round. They showed him a video of himself and what had happened. He was completely shocked he had no idea how it had affected him and those around him. The video showed him on the floor with the paddles attached. Some members of staff on the video were visibly upset. David decides that this must stop but he doesn't know where to start.

Shane is 17 years old he has been convicted of robbery when he got involved with a gang of youths. He used to live with his grandma and dad who come to visit him regularly. One week his dad comes along to visit him without his grandma to tell him that his grandma has suddenly died from a heart attack. Shane was devastated he did not know how to handle the news – he goes back to his cell and wrecks his pad – and he is taken to the segregation unit to calm down. When he returns to the wing Shane is determined to get himself back on track, he is worried about whether he will be able to attend the funeral and what he can do to support his Dad.

Joseph an older man worked in banking industry for 25 years. He was finally convicted for fraud after a lengthy case and Joseph was sent to prison for 9 years. He was shocked by his incarceration and having been in Leeds for a few months he has been transferred to another prison, but he has struggled to adjust to prison life. He feels completely out of place. He looks like he is not looking after himself – his son comes to visit – he is ashamed of his father's behaviour and tells him he does not want to be part of his life anymore. A member of staff identifies Joseph as needing some help and refers him to you to see how best Joseph can be supported. Joseph feels isolated, alone, and angry, he refuses to come out of his cell when asked to by a member of staff.

For peer review only

Before Patrick came into prison my car was stolen. Patrick was recalled into prison on licence for not following the rules. On the night before he was arrested, he was involved in a car incident which meant that my car was stolen. He doesn't know what has happened to it. He is still paying road tax – He doesn't know if it is still OK or what to do about the insurance. He needs his car on the outside because he is a taxis driver – He is worried that when he is released, he won't be able to work and he won't have any money. He is talking to other prisoners to see if 'one of the boys' on the outside might be able to help, but the problem seems too big he wants to ignore it and pretend it hasn't happened. He thinks he can do nothing about his problem whilst in prison.

Andrew has a long history of self-harm behaviour and he has been monitored by staff for his self-harm behaviour for many months. Andrew has self-harmed himself ever since being in prison. Andrew is quite quiet and generally keeps himself to himself – he has a family and two children outside and he hasn't been able to pay the mortgage on the house for two months. The families' savings have all run out. Last week his wife came to visit in tears saying that she was worried that the house would be re-possessed. This is Andrew's main concern now. He feels completely out of control, he is panicking about what he can do to help support his family and his wife. He doesn't have a clear plan in is head about how he can help to resolve the situation and is feeling very anxious he feels like he needs to advise his wife on what they should do but thinks that time is running out for them. He blames himself for the situation and has been more reluctant to come out of his cell and go to work.

For peer review only

Consolidated criteria for reporting qualitative studies (COREQ): 32-item checklist

No	Item	Guide questions/description	Page numbers where marked in manuscript
**Domain 1: Research team and reflexivity**			
Personal Characteristics			
1.	Interviewer/facilitator	Which author/s conducted the interview or focus group?	Author MZ
2.	Credentials	What were the researcher's credentials? *E.g. PhD, MD*	Page 7
3.	Occupation	What was their occupation at the time of the study?	Page 7
4.	Gender	Was the researcher male or female?	Inferred by use of initials; MZ on page 7
5.	Experience and training	What experience or training did the researcher have?	Page 7 inferred by reference to qualification
Relationship with Participants			
6.	Relationship established	Was a relationship established prior to study commencement?	Page 6 under sub-heading ‘study collaboration’
7.	Participant knowledge of the interviewer	What did the participants know about the researcher? e.*g. personal goals, reasons for doing the research*	Not applicable
8.	Interviewer characteristics	What characteristics were reported about the interviewer/facilitator? e.g. *Bias, assumptions, reasons and interests in the research topic*	None reported
**Domain 2: study design**			

No	Item	Guide questions/description	Page numbers where marked in manuscript
Theoretical framework			
9.	Methodological orientation and Theory	What methodological orientation was stated to underpin the study? *e.g. grounded theory, discourse analysis, ethnography, phenomenology, content analysis*	Page 8 and see Reference 30.
Participant selection			
10.	Sampling	How were participants selected? *e.g. purposive, convenience, consecutive, snowball*	Pages 7-8
11.	Method of approach	How were participants approached? e.*g. face-to-face, telephone, mail, email*	Pages 7-8
12.	Sample size	How many participants were in the study?	Pages 9-10 and in the Abstract
13.	Non-participation	How many people refused to participate or dropped out? Reasons?	Page 8 and under sub-heading 'stage four….'
Setting			
14.	Setting of data collection	Where was the data collected? e.*g. home, clinic, workplace*	Pages 7-8 and in the Abstract
15.	Presence of non-participants	Was anyone else present besides the participants and researchers?	No-one else was present.
16.	Description of sample	What are the important characteristics of the sample? *e.g. demographic data, date*	Pages 9-10
Data collection			
17.	Interview guide	Were questions, prompts, guides provided by the authors? Was it pilot tested?	Use of semi-structure self-report surveys were devised by the research team

No	Item	Guide questions/description	Page numbers where marked in manuscript
18.	Repeat interviews	Were repeat interviews carried out? If yes, how many?	No
19.	Audio/visual recording	Did the research use audio or visual recording to collect the data?	No
20.	Field notes	Were field notes made during and/or after the interview or focus group?	No
21.	Duration	What was the duration of the interviews or focus group?	Page 7 – A 90 minute session of instruction was used with staff and students in preparation for completion of the survey.
22.	Data saturation	Was data saturation discussed?	No
23.	Transcripts returned	Were transcripts returned to participants for comment and/or correction?	Not applicable
**Domain 3: analysis and findings**			
Data analysis			
24.	Number of data coders	How many data coders coded the data?	Two see page 8
25.	Description of the coding tree	Did authors provide a description of the coding tree?	No
26.	Derivation of themes	Were themes identified in advance or derived from the data?	Themes were not derived in advance but from the data.
27.	Software	What software, if applicable, was used to manage the data?	Page 8 see sub-heading 'Data Analysis'. Reference to Excel software

No	Item	Guide questions/description	Page numbers where marked in manuscript
28.	Participant checking	Did participants provide feedback on the findings?	Participants were part of the consultation process and provided feedback on the adapted package see stage four of the project.
Reporting			
29.	Quotations presented	Were participant quotations presented to illustrate the themes / findings? Was each quotation identified? e*.g. participant number*	Yes, quotations of text are used to illustrate examples of the themes
30.	Data and findings consistent	Was there consistency between the data presented and the findings?	Yes
31.	Clarity of major themes	Were major themes clearly presented in the findings?	Table 2; see also within the text on pages 9-10
32.	Clarity of minor themes	Is there a description of diverse cases or discussion of minor themes?	No, diverse cases or discussion of minor themes were reported.

only
